# Dual SLIPT–A Lipid Mimic to Enable Spatiotemporally Defined, Sequential Protein Dimerization

**DOI:** 10.1021/acschembio.4c00856

**Published:** 2025-04-15

**Authors:** Kristina V. Bayer, Maedeh Taeb, Birgit Koch, Shige H. Yoshimura, Richard Wombacher

**Affiliations:** † Department of Chemical Biology, Max Planck Institute for Medical Research, Jahnstraße 29, 69120 Heidelberg, Germany; ‡ Heidelberg Biosciences International Graduate School (HBIGS), Heidelberg University, Im Neuenheimer Feld 501, 69120 Heidelberg, Germany; § Graduate School of Biostudies, Kyoto University, Kyoto 606-8501, Japan; ∥ Center for Living Systems Information Science (CeLiSIS), Kyoto University, Kyoto 606-8501, Japan; ⊥ Institute for Integrated Cell-Material Sciences (iCeMS), Kyoto University, Kyoto 606-8501, Japan

## Abstract

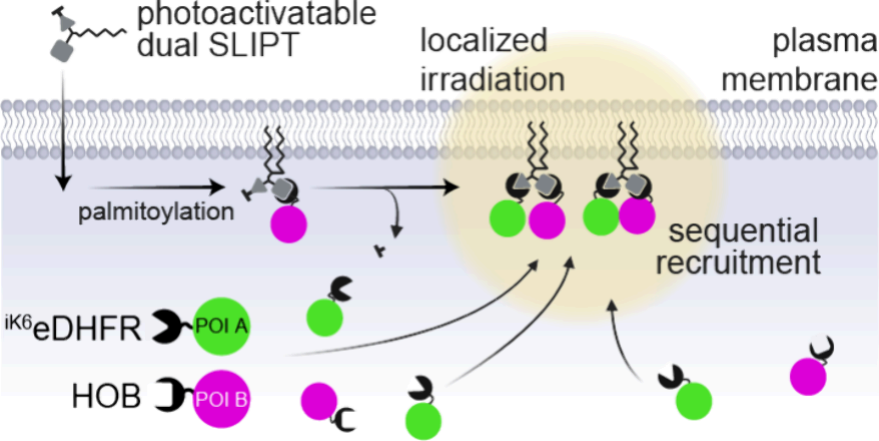

Spatiotemporal control of proteins is crucial for cellular phenomena such as signal integration, propagation, as well as managing crosstalk. In membrane-associated signaling, this regulation is often enabled by lipids, wherein highly dynamic, sequential recruitment of interacting proteins is key to successful signaling. Here, we present dual SLIPT (self-localizing ligand-induced protein translocation), a lipid-analog tool, capable of emulating this lipid-mediated sequential recruitment of any two proteins of interest. Dual SLIPT self-localizes to the inner leaflet of the plasma membrane (PM). There, dual SLIPT presents trimethoprim (TMP) and HaloTag ligand (HTL) to cytosolic proteins of interest (POIs), whereupon POIs fused to the protein tags ^iK6^eDHFR, or to HOB are recruited. A systematic extension of the linkers connecting the two mutually orthogonal headgroups was implemented to overcome the steric clash between the recruited POIs. Using Förster resonance energy transfer (FRET), we verify that the resulting probe is capable of simultaneous binding of both proteins of interest, as well as their dimerization. Dual SLIPT was found to be particularly suitable for use in physiologically relevant concentrations, such as recruitment via tightly regulated, transient lipid species. We further expanded dual SLIPT to the photocontrollable dual SLIPT^NVOC^, by introducing a photocaging group onto the TMP moiety. Dual SLIPT^NVOC^ enables sequential and spatiotemporally defined dimerization upon blue light irradiation. Thus, dual SLIPT^NVOC^ serves as a close mimic of physiology, enabling interrogation of dynamic cytosol-to-plasma membrane recruitment events and their impact on signaling.

## Introduction

Cellular spatiotemporal control of proteins is a key determinant of protein function in signaling.^1^ Crucial phenomena such as signal integration, propagation, and crosstalk are managed by the sequential recruitment of higher-order protein assemblies. The plasma membrane (PM), as the locus for such signaling sequences, is of particular interest. At the PM, transient lipid-mediated recruitment of proteins can abruptly increase a protein’s effective local concentration.^2^ Conversely, degradation of specific lipids can reverse protein localization at the PM, enabling dynamic control over protein availability. Similarly, protein–lipid specificity enables lateral organization of interactors.^3,4^ Thus, lipid-mediated PM-recruitment can enable dynamic spatiotemporally defined compartmentation and organize signaling.^5^

During chemotaxis, a well-studied process in which lipid-mediated recruitment underpins functional outcomes, spatiotemporal signal integration between GPCR–Ras activation and directed cell movement (mediated by Rac^6^) is required. After GPCR activation, active Ras recruits phosphatidylinositol 3-kinase (PI3K) to the leading edge of the PM. There, PI3K generates phosphatidylinositol triphosphate (PIP_3_) species, capable of recruiting actin modifiers such as Rac, which subsequently induce directional cellular movement.^5^

This underscores the importance of elucidating recruitment sequences to understand cause-and-effect in signaling pathways. Investigating localized interactions and their impacts on signaling has been investigated in various ways. Among these, protein-based tools offer powerful means of external, precise manipulation.

Among protein-based tools, optogenetics^7−9^ and chemogenetics^10−14^ find widespread application. Optogenetics benefit from ease of accessibility. They require, however, case-by-case optimization to maximize the difference between dark and light states,^15^ as well as continuous irradiation.^16^ Chemogenetics, or chemical inducers of dimerization (CIDs), on the other hand, are extraordinarily useful due to their “plug-and-play” nature: protein proximity is induced by adding a small molecule that forces together any two proteins fused to the appropriate tags.

Protein-based tool approaches require one of the partners to be prelocalized, if investigating localized signaling that depends on the recruitment of multiple proteins of interest (POIs) and their interaction at a specific subcellular location.^17,18^ Upon the dimerization stimulus (irradiation or addition of the dimerizing chemical), then, only a single POI changes localization. Thus, these approaches confine the investigation to cellular responses that can accommodate prolonged prelocalization of a single POI.

Alternatively, two tags can be expressed to translocate a single POI. As signaling rarely only involves translocation of a single POI, strategies for recruiting multiple POIs are required. The first chemically inducible trimerization (CIT) system^19^ can be used to dimerize two POIs at a specific location. However, this approach, too, requires the expression of more protein tags than POIs to be translocated.

While useful, recent interest has moved away from protein-based tools toward tools with fewer, or minimal, less sterically demanding tags.^20,21^ The ideal tool to translocate one or more POIs to a specific subcellular localization would encompass a localization motif that is not reliant on a bulky protein tag.^22^ One such example is the chemogenetic control of protein localization in mammalian cells by self-localizing ligand-induced protein translocation (SLIPT).^23−28^ First reported in 2013, the SLIPT family of probes enabled recruitment of any POI to the nucleus, microtubuli, the Golgi, the ER, as well as the PM. The “self-localizing” nature of the SLIPT system is based on small molecules that localize to the specific cellular target. There, SLIPT probes present a “headgroup”-moiety, by which client POIs can be recruited. This renders them a one probe–one protein-tag system. The headgroup can be varied to recruit any POI, tagged with either eDHFR, SNAP, or HT7.^25^ These protein tags are orthogonal to the mammalian proteome, which avoids unwanted cross-reactivity.

**Figure 1:**
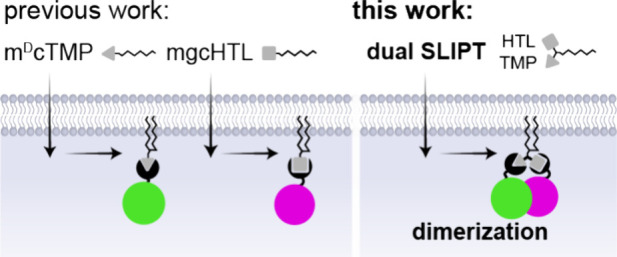
Monomeric SLIPT systems (left) compared to dual SLIPT (right). Monomeric versions m^D^cTMP and mgcHTL are recruiting ^iK6^eDHFR or HOB fused proteins of interest, respectively. Dual SLIPT, conversely, recruits both ^iK6^eDHFR and HOB-tagged proteins of interest. Upon permeation into the cell, SLIPT probes are palmitoylated, adding another lipid to the localization motif. HTL, HaloTag ligand; TMP, Trimethoprim.

If the aim is to emulate lipid-mediated translocation of proteins to the PM, lipid-analog tools that are capable of POI recruitment would be ideal. In analogy to endogenous lipids, PM-SLIPT^23^ targets the PM through a lipid motif (Figure 1). Thus, PM-SLIPT, in particular, closely mimics physiological protein recruitment by lipids.

In this work, we created a tool for interrogating lipid-mediated cytosol-to-PM recruitment events and their impacts on signaling. To this end, we aimed at recruiting more than just one POI to the PM. Based on the SLIPT concept, we generated dual SLIPT. Dual SLIPT is a PM-localizing lipo-peptide tool capable of chemically dimerizing any two POIs at the inner leaflet of the PM in a spatiotemporally defined, sequential manner (Figure 1). We present the design and engineering of dual SLIPT, which allows for both interrogation and manipulation of signaling hierarchies, thus expanding the repertoire of self-localizing probes.

We synthesized a series of compounds to dimerize a mutually orthogonal set of protein tags: ^iK6^eDHFR (an eDHFR variant, optimized for PM-recruitment^28^) and Halo-based Oligonucleotide Binder (HOB^11^). By optimizing the linker lengths between the two headgroups (TMP and HTL), we successfully generated dual SLIPT. Dual SLIPT is a self-localizing lipid-based tool capable of simultaneously binding and thus enforcing interaction between two POIs. Dual SLIPT retains the plug-and-play aspect of conventional CIDs, as any POIs can be genetically fused to the two protein tags. Additionally, we exploited its modular design to incorporate photocaging of one headgroup (TMP), enabling greater spatiotemporal precision and sequential recruitment. This lipid-like tool sequentially recruits and dimerizes two POIs, facilitating the study of signaling hierarchies and their effects on signaling outcomes.

## Results and Discussion

### Design of a SLIPT Variant Capable of Recruiting Two Different Proteins to the PM

We intended this study to generate a new self-localizing ligand system, which we call **dual SLIPT**. **Dual SLIPT** targets the inner leaflet of PM and is capable of dimerizing any two POIs in a mutually orthogonal manner. This chemogenetic tool was intended to enable biological investigations of the impact of temporal sequence on PM-localized dimerization events on cellular signaling.

**Figure 2:**
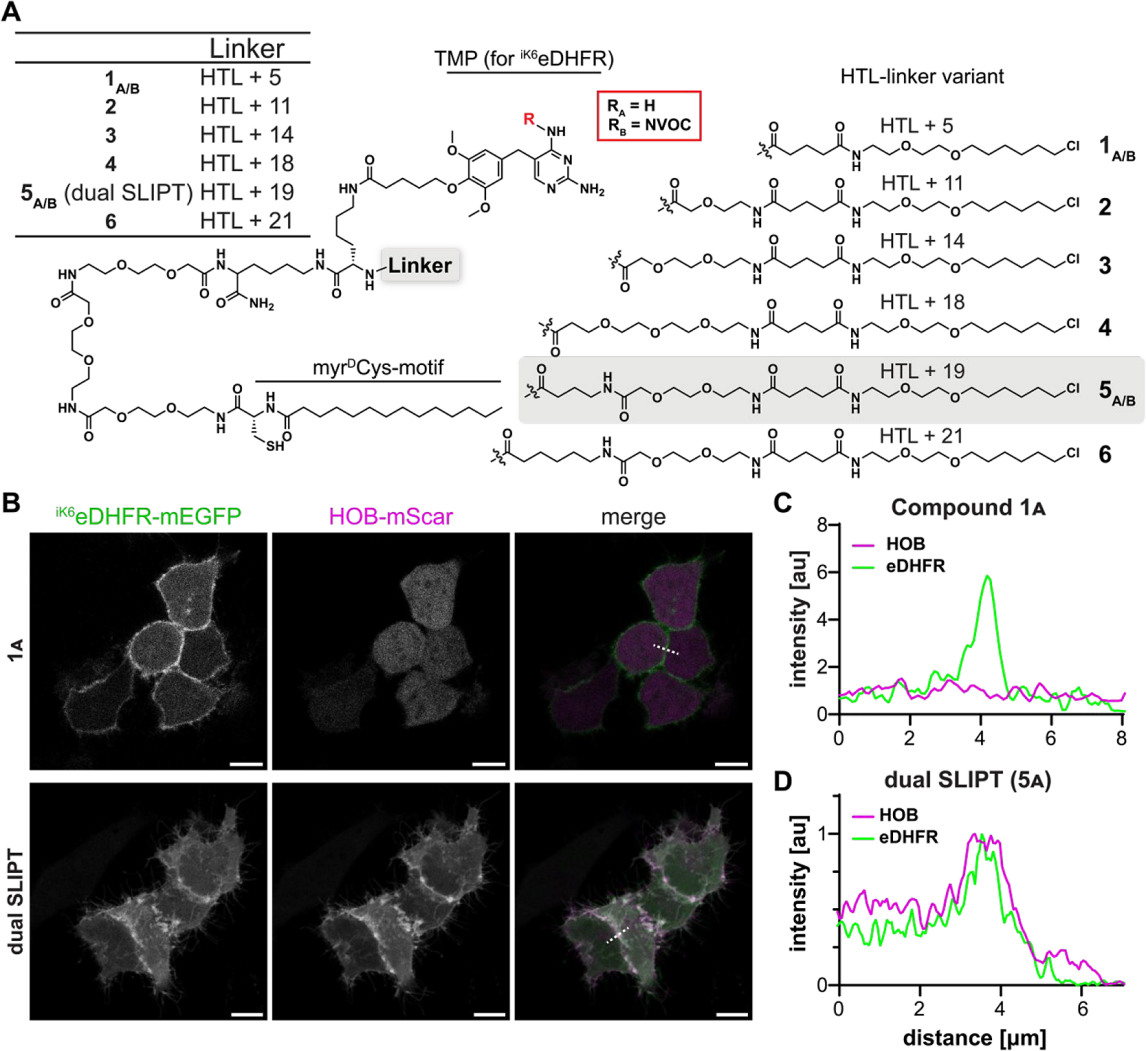
Design and optimization of dual SLIPT. (A) Chemical structures of trivalent SLIPT probes used in this study and a table delineating the linker lengths spanning HTL-amine to the remaining linker. Numbers are indicative of number of atoms. Shaded compound (5_A_) depicts dual SLIPT, the first probe in this series, capable of dual recruitment. NVOC = 6-nitroveratryloxycarbonyl; Subscript A refers to nonphotocaged, and B to NVOC-caged variants. (B) HeLa cells expressing ^iK6^eDHFR-mEGFP (green) and HOB-mScar (magenta), incubated with 10 μM m^D^cTMP-HTL^5^, (1_A_, top), or dual SLIPT (m^D^cTMP-HTL^19^, 5_A_, bottom), overnight. (C) Line plots depicting a representative cross section of fluorescent intensities across the plasma membrane of two adjacent cells that are incubated with 10 μM (1_A_). eDHFR, ^iK6^eDHFR-mEGFP (green); HOB, HOB-mScar. (D) Line plots depicting a representative cross section of fluorescent intensities across the plasma membrane of two adjacent cells that are incubated with 10 μM dual SLIPT, (5_A_). Scale bar, 10 μm.

To do so, we chose the ^iK6^eDHFR-tag and HaloTag7 (HT7) as starting points for protein tags. We chose the myristic acid d-cysteine (m^D^c) motif, wherein both myristic acid and the in cellulo palmitoylation of cysteine target the probe to the PM’s inner leaflet. The unnatural D-configuration protects the probe from proteolytic degradation and loss of PM-localization over time.^24,27,29^ Three repeats of 8-Amino-3,6-dioxaoctanoic acid (a PEG2-unit) act as spacers between headgroups and PM-inserting lipids, as optimized in the existing family of SLIPTs.^23^ The PM-inserting motif is connected to the headgroups via the branching amino acid lysine, rendering dual SLIPT a trivalent chemogenetic. Guided by the existing literature on TMP–HT7-based chemical inducers of dimerization (CIDs),^30^ we began by synthesizing self-localizing compound **1**_**A**_ (**m**^**D**^**cTMP-HTL**^**5**^, Figure 2A), with minimal linkers between the two headgroups TMP and HTL, as a starting point.

### Optimizing the Linker to Enable Dual Recruitment of ^iK6^eDHFR and HOB to the PM

To assess whether simultaneous recruitment of ^iK6^eDHFR and HT7 to the PM was possible via compound **1**_**A**_, HeLa cells expressing both ^iK6^eDHFR fused to monomeric enhanced green fluorescent protein (mEGFP) and HT7 fused to mScarlet (mScar) were incubated with 10 μM of **1**_**A**_ for 1h in DMEM(−). Translocation was assessed in live cells, using confocal fluorescent imaging. Substantial translocation of ^iK6^eDHFR-mEGFP to the PM occurred (Figure S1A, top). Incubation with **1**_**A**_, however, failed to translocate HT7-mScar alongside ^iK6^eDHFR-mEGFP in the given time frame. As mgcHTL^25^ had previously induced HT7–to–PM translocation within 30 min, and ^iK6^eDHFR-recruitment indicated probe presence in the inner leaflet, we determined the incubation duration was sufficient to conclude that compound **1**_**A**_ is unable to induce dual recruitment of ^iK6^eDHFR-mEGFP and HT7-mScar.

We reasoned that simultaneous recruitment of both POIs was failing due to one of the two following factors. Either the distance of the reactive chloroalkane from the membrane was too short to be accessible to HT7-mScar, or steric clash between the two POIs prevented simultaneous binding. We assumed lacking accessibility to be the cause for two reasons: Previously described dimerizers based on TMP and HTL with comparable distances between the two ligands, as well as a SLIPT recruiting HT7 to the PM with five PEG2-repeats, were already reported.^20,25,30^

To rule out steric clash between ^iK6^eDHFR-mEGFP and HT7-mScar as the cause of failed simultaneous recruitment, we synthesized the photocaged derivative of **1**_**A**_ (**m**^**D**^**cTMP**^**NVOC**^**-HTL**^**5**^, **1**_**B**_), which contains a photocaged TMP, preventing initial ^iK6^eDHFR-mEGFP recruitment to the PM (Figure S1D). This would allow assessment of whether the chloroalkane ligand was accessible to HT7-mScar in the absence of ^iK6^eDHFR-mEGFP. To do so, we incubated HeLa cells, expressing ^iK6^eDHFR-mEGFP and HT7-mScar, with 10 μM of **1**_**B**_ for 1h in DMEM(−) and assessed HT7-mScar translocation. Despite the absence of probe-associated ^iK6^eDHFR-mEGFP, HT7-mScar translocation was not observed after incubation with **1**_**B**_ (Figure S1C, top). To verify whether **1**_**B**_ is capable of permeating the cell and localizing correctly in the inner leaflet, the TMP moiety of **1**_**B**_ was uncaged by irradiation with 405 nm light, which successfully led to immediate recruitment of ^iK6^eDHFR-mEGFP to the PM, indicating correct localization of the probe. This led us to consider the impact of electrostatic repulsion between the binding interface of HT7-mScar and the negatively charged surface of the inner leaflet. Thus, we switched to Halo-based Oligonucleotide Binder (HOB^11^), to assess whether this variant of HT7-mScar was able to be recruited to the inner leaflet. HOB has positively charged amino acids surrounding the binding-cleft, which has been shown to be crucial in improving binding rates for negatively charged substrates.^11,31^

HeLa cells expressing both HOB-mScar and ^iK6^eDHFR-mEGFP showed translocation of HOB to the PM, after incubating with the photoactivatable probe **1**_**B**_ for 1 h (Figure S1C, bottom). However, after irradiation with light, thereby uncaging the TMP ligand in **1**_**B**_, we did not now observe recruitment of ^iK6^eDHFR-mEGFP to the PM. This showed that while HOB, unlike HT7, can be recruited to the membrane, steric hindrance prevents dual recruitment of both protein tags (HOB and ^iK6^eDHFR). Interestingly, this is confirmed when HeLa cells expressing ^iK6^eDHFR-mEGFP and HOB-mScar were treated with **1**_**A**_. Now, due to the faster binding kinetics of ^iK6^eDHFR-mEGFP to the probe, subsequent HOB-mScar recruitment was prevented (Figure 2B, top; Figure 2C; S1A, bottom). This led us to the realization that, while the recruitment of protein tags is possible in principle, steric hindrance precluded dual recruitment. Thus, to attain the functional probe **dual SLIPT**, we began systematically lengthening the linker that connects the headgroups.

### Increasing Linker Lengths between HTL and TMP Leads to Dual Recruitment of ^iK6^eDHFR-mEGFP and HOB-mScar

Since switching from HT7 to HOB as the protein tag successfully overcame the electrostatic repulsion between the tag and the inner leaflet, we next addressed the steric clash that was preventing dual recruitment of both ^iK6^eDHFR-mEGFP and HOB-mScar. We chose to synthesize a series of self-localizing probes (**1**_**A**_, **2–4**, and **5**_**A**_, Figure 2A), with progressively increasing linker lengths between HTL and TMP. PEG units and alkyl chains, connected through amide bonds, were chosen as spacers due to their synthetic accessibility and solubility. The ability of compounds **2–4** and **5**_**A**_ to translocate both ^iK6^eDHFR-mEGFP and HOB-mScar was assessed in live cells. HeLa cells stably expressing HOB-mScar, as well as ^iK6^eDHFR-mEGFP, were incubated with 10 μM of the respective compound in DMEM(−) overnight. Fluorescent protein localization was analyzed via confocal imaging the next day.

While compounds **1**_**A**_ and **2–4** showed ^iK6^eDHFR-mEGFP translocation in cells expressing both POIs (Figure 2B top, C; Figure S2), simultaneous HOB-mScar-to-PM recruitment was not observed. Notably, in cells expressing only HOB-mScar, recruitment of this POI to the PM was observed, further demonstrating that HTL-linker lengths between 5 and 18 atoms were only capable of single recruitment. However, with **5**_**A**_ (**m**^**D**^**cTMP-HTL**^**19**^, **dual SLIPT**), recruitment of both HOB-mScar, as well as ^iK6^eDHFR-mEGFP was observed (Figure 2B bottom, D). Dual recruitment was also observed with **6** (**m**^**D**^**cTMP-HTL**^**21**^), which has a linker length exceeding that of **5**_**A**_ (Figure S2). However, translocation kinetics induced by **6** did not show any improvements compared to **5**_**A**_. Thus, we chose **5**_**A**_ for further experiments.

A comparison of **4** (**m**^**D**^**cTMP-HTL**^**18**^) and **5**_**A**_ (**dual SLIPT**), which differ in the chain length by a single −CH_2_ and in composition (Figure 2A), revealed a significant increase in PM translocation of HOB-mScar alongside ^iK6^eDHFR-mEGFP with **5**_**A**_. This finding could indicate that **5**_**A**_ contains the minimal linker length required to recruit both ^iK6^eDHFR-mEGFP and HOB-mScar using a single molecule of probe. However, factors such as linker rigidity, solvation, conformation, or beneficial interactions introduced by the amide bond could very well also contribute to the ability of **5**_**A**_ to dually recruit both POIs.

Previously published bifunctional CIDs of eDHFR and HT7^30^ reported shorter optimal distances between TMP and HTL headgroups than observed in this study. This indicates a synergistic effect of the optimizations implemented here, addressing the electrostatic repulsion with the inner leaflet and the steric demand of the protein tags themselves.

### **Dual SLIPT** Is Capable of Dimerizing ^iK6^eDHFR- and HOB-Fused POIs

Next, we wanted to confirm that a single molecule of **dual SLIPT** is able to recruit both protein tags and thus is capable of dimerizing two target proteins at the PM. Dimerization can be distinguished from mere colocalization to the PM by an enforced interaction between the two POIs fused to the protein tags. Thus, we evaluated dimerization induced by **dual SLIPT**, as opposed to mere colocalization, in live cells. To do so, we chose Förster Resonance Energy Transfer (FRET) between two fluorescent proteins fused to ^iK6^eDHFR and HOB.

**Figure 3:**
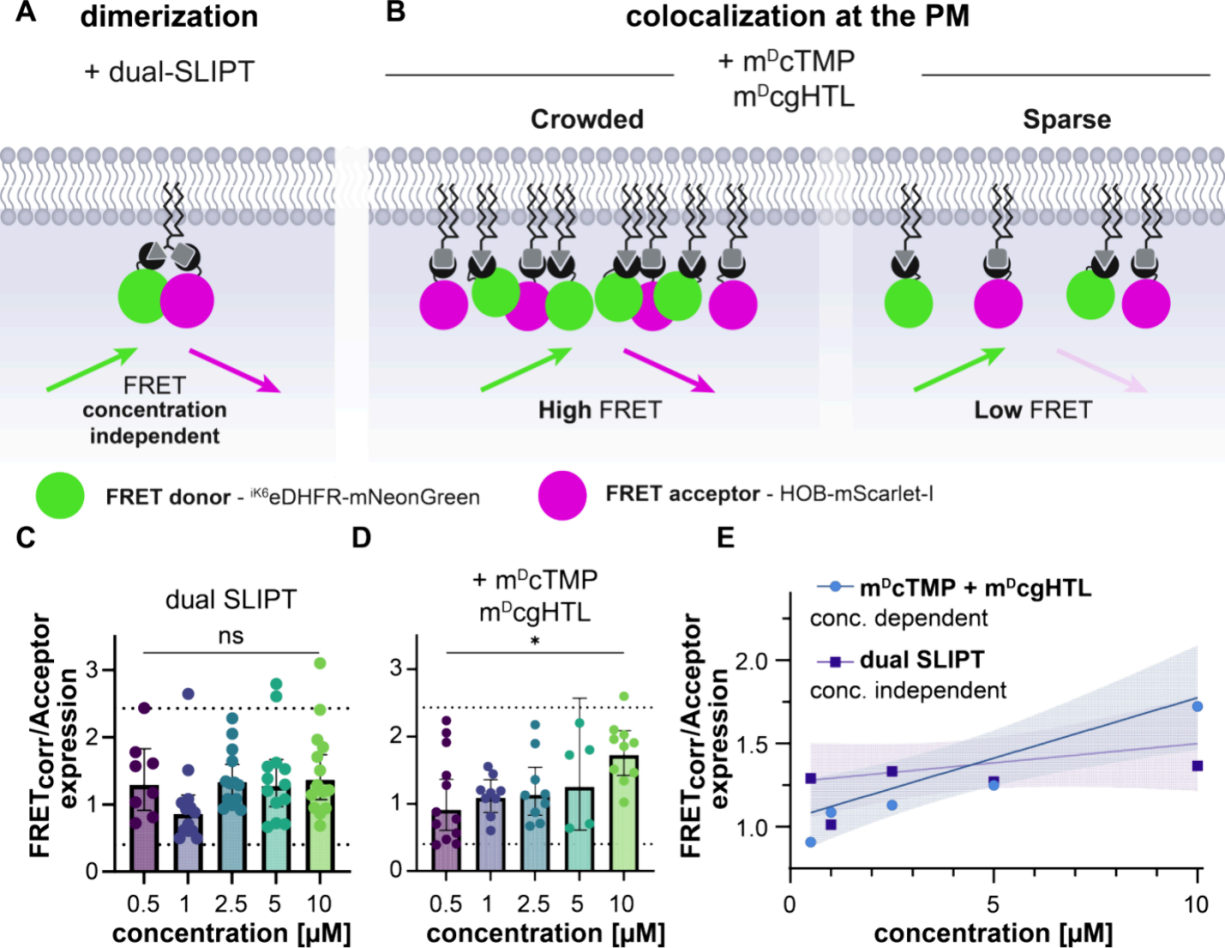
One molecule of dual SLIPT can bind both POIs, via their respective protein tags, simultaneously. (A) Schematic illustration of the concept behind this FRET experiment. Addition of dual SLIPT leads to simultaneous recruitment of ^iK6^eDHFR-tagged FRET donor (green), and HOB-tagged FRET acceptor (magenta). As the mean distance between donor and acceptor is constant, irrespective of probe concentration, FRET efficiency should also be constant, irrespective of probe concentration. (B) Schematic illustration to compare the control system. Addition of equal concentrations of m^D^cTMP (recruiting ^iK6^eDHFR-tagged FRET donor) and m^D^cgHTL (recruiting HOB-tagged FRET acceptor), also recruit the FRET pair to the PM. However, as in this control system each probe recruits an individual POI, the mean distance between donor and acceptor should increase with lowered probe concentration. This leads to concentration dependent FRET. (C) FRET_CORR_ normalized to acceptor expression in dependence of dual SLIPT concentration (quantification of schematic A). HeLa cells incubated with dual SLIPT (5_A_) showed a median FRET_CORR_ value of 1.29 at 0.5 μM 5_A_ (*n* = 8 fields of view, 9684 ROIs) and 1.37 at 10 μM 5_A_ in DMEM(−) (*n* = 14 fields of view, >15,000 ROIs), (*p* > 0.05, Mann–Whitney test). Each data point represents an averaged value of a field of view, with multiple cells, each. (*n* > 5000 ROIs per condition); dotted lines represent minimal and maximal possible FRET with this FRET pair, respectively. (D) FRET_CORR_ normalized to acceptor expression in dependence of mixed m^D^cTMP and m^D^cgHTL concentrations (quantification of schematic B). The probe mixture of **7** and **8**, induced median FRET_CORR_ of 0.91 at 0.5 μM (*n* = 11 fields of view, >15,000 ROIs) and 1.72 at 10 μM (*n* = 10 fields of view, >10,000 ROIs), (*p* = 0.0249, Mann–Whitney test). Each data point represents an averaged value of a field of view, with multiple cells, each (*n* > 5000 ROIs per condition). (E) Median FRET_CORR_ normalized to acceptor expression are plotted against m^D^cTMP and m^D^cgHTL concentration (blue), and dual SLIPT concentration (purple), and fitted linearly. Although both positive, only the fitted slope of FRET efficiency induced by m^D^cTMP and m^D^cgHTL significantly deviated from zero (*p* = 0.0013). The slope’s deviation from zero induced by dual SLIPT did not reach significance (*p* = 0.2823, ns).

Although accepted as a measure of protein interaction,^32^ FRET, by itself, is not a measure of dimerization. As FRET efficiency decreases with the sixth power of the radius between the two fluorophores,^33^ it is a measure of distance. However, we reasoned that if **dual SLIPT** were capable of dimerization, the mean distance between the two fluorescent proteins should remain constant, regardless of the **dual SLIPT** concentration. Thus, the FRET efficiency between donor and acceptor should also remain constant (Figure 3A).

As a negative control, we chose a system that is incapable of dimerization but enables localization of both target proteins to the PM. To this end, the previously reported SLIPT m^D^cTMP (**7**)^24^ and m^D^cgHTL (**8**, see SI), which can each recruit a single protein tag, were mixed. As this combination of probes can “merely” colocalize, this experimental condition relies on crowding at the PM for FRET. The FRET pair recruited by the mixture of **7** and **8** would show high FRET at high probe concentrations, causing crowding of the donor and acceptor at the PM. Conversely, low FRET is expected when the concentration of the probes is low (Figure 3B).

To assess the relationship between FRET efficiency and probe concentration in the two experimental setups (Figure 3A vs B), we fused ^iK6^eDHFR to mNeonGreen (mNG, FRET donor) and HOB to mScarlet-I (mScar-I, FRET acceptor). HeLa-cells expressing these constructs were incubated overnight with either **dual SLIPT** (**5**_**A**_) [0.5–10 μM] (Figures 3C and S3E), or m^D^cgHTL (**8**) [0.5–10 μM] in DMEM(−). To the m^D^cgHTL-treated cells, we added equal concentrations of **7**, 30 min prior to imaging (Figure 3D and S3D).

As a positive control, we generated a tandem construct of ^iK6^eDHFR fused to both donor and acceptor, including a previously reported optimized linker,^34^ to maximize FRET efficiency. HeLa cells expressing the tandem ^iK6^eDHFR-mNG-mScar-I fusion construct were then treated with **7** in DMEM(−) 30 min prior to imaging [10 μM] (S3A–C). To determine the minimal possible (bystander) FRET,^35,36^ we expressed ^iK6^eDHFR -mNG and HOB-mScar-I, and added only **7** [10 μM] in DMEM(−) 30 min prior to imaging, to recruit the FRET-donor (mNG) “away” (to the PM) from the FRET-acceptor (mScar-I, cytosol). The difference between maximal and minimal FRET_CORR_ was determined to be 6.05-fold (*p* < 0.0001).

HeLa cells expressing the donor and acceptor pair separately, and incubated with **dual SLIPT** (**5**_**A**_), showed a median FRET_CORR_ value of 1.29 at 0.5 μM and 1.37 at 10 μM. This difference was insignificant, which means that FRET efficiency induced by **dual SLIPT** (**5**_**A**_), and thus distance between the recruited POIs, is independent of concentration. In contrast, the probe mixture of **7** and **8**, relying on crowding to induce FRET, induced a median FRET_CORR_ value of 0.91 at 0.5 μM and 1.72 at 10 μM, which is significant (*p* = 0.0249). This dependence on probe concentration indicates that the median distance between the recruited POIs increases as probe concentration decreases. This confirms that, unlike **dual SLIPT**, the mixture of **7** and **8** is unable to induce dimerization and merely colocalizes the recruited POIs at the PM.

To further illustrate this difference of **dual SLIPT-induced** dimerization to colocalization at the PM, we plotted FRET_CORR_ linearly against the respective probe concentrations. The slopes, although both positive, only significantly deviated from zero in the case of the probe mixture (**7** and **8**) (*p* = 0.0013). The slope’s deviation from zero induced by **dual SLIPT** did not reach significance (*p* = 0.2823, ns), consolidating that the FRET efficiency induced by **dual SLIPT** is independent of probe concentration. This further confirms that **dual SLIPT** binds both ^iK6^eDHFR and HOB and enforces dimerization between the fused POIs.

### Photoactivatable Dual SLIPT^NVOC^, Capable of Spatially and Temporally Defined, Sequential Dimerization at the PM with Single Cell Resolution

Having demonstrated that **dual SLIPT** is capable of dimerization, we sought to expand the system to gain spatial and temporal control over the dimerization event via light control. This is particularly important for the investigation of dynamic recruitment events to limit the time for compensatory mechanisms, improving spatial control, or ensuring same-well negative controls.

**Figure 4:**
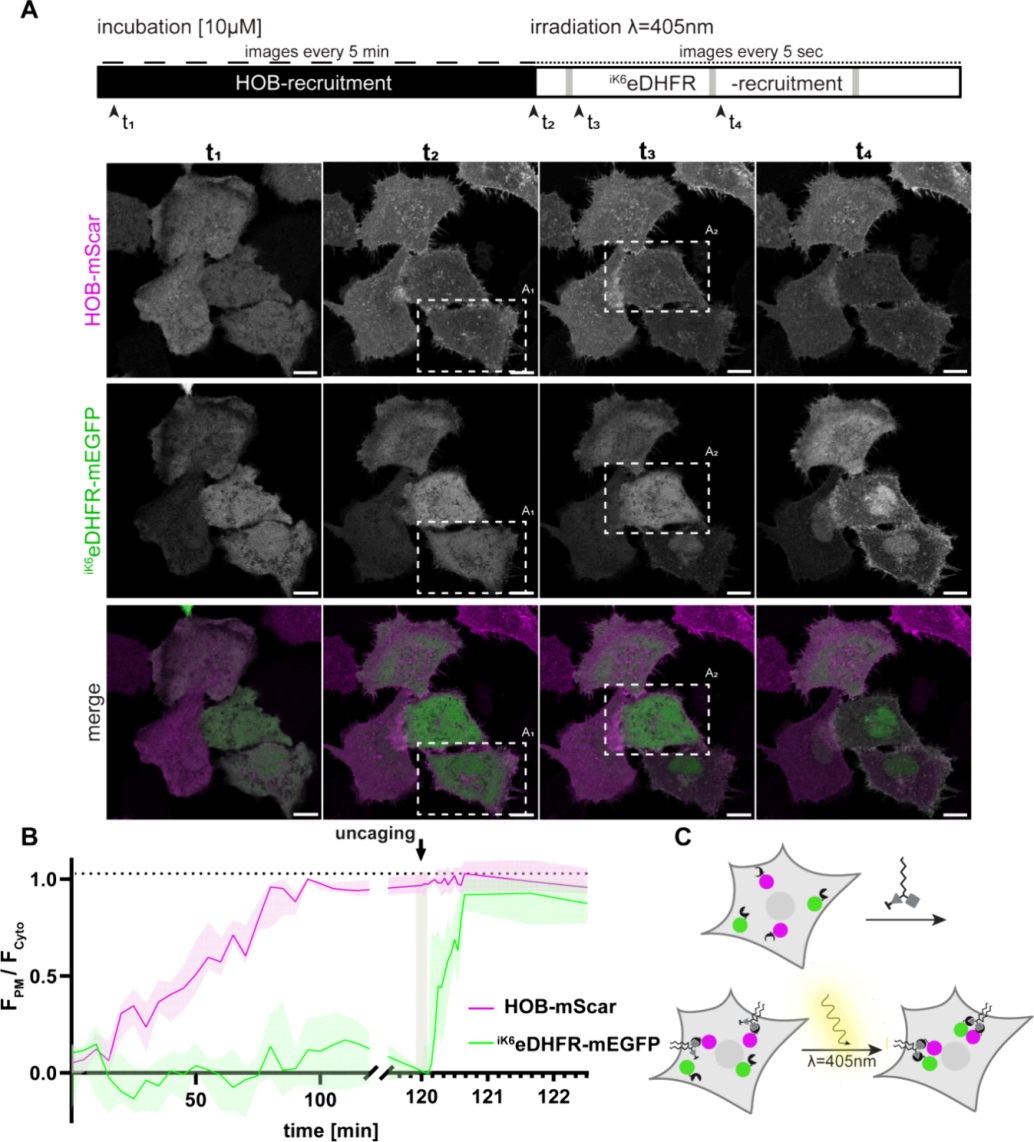
Dual SLIPT^NVOC^enables sequential recruiting and spatiotemporally defined control over the dimerization event. (A) Schematic depicting the imaging conditions, and expected cyto-to-pm recruitment events, as well as representative live cell data. Incubation with dual SLIPT^NVOC^ translocates HOB-mScar first, before irradiation with 405 nm cleaves off the photocaging group on TMP, which thereafter rapidly recruits ^iK6^eDHFR-tagged POIs. HeLa cells expressing ^iK6^eDHFR-mEGFP (green) and HOB-mScar (magenta), incubated with 10 μM dual SLIPT^NVOC^ (m^D^cTMP^NVOC^-HTL^19^) while under observation. t_1_ indicates the preincubation image, t_2_ indicates maximal HOB translocation (preirradiation), t_3,4_ indicate postirradiation images of regions of interest indicated with dashed lines. Scale bar, 10 μm. (B) Quantitation of the cyto-to-pm recruitment plotted against time of HOB-mScar (magenta) and ^iK6^eDHFR-mEGFP (green). F_PM_, Fluorescence intensity at the plasma membrane; F_Cyto_, fluorescence intensity in the cytoplasm. (C) Schematic depicting the recruitment sequence in response to dual SLIPT^NVOC^ addition.

The affinity of eDHFR for TMP can be suppressed by the introduction of a photocaging group on the exocyclic amines of TMP.^37^ Removal of the photocaging group by irradiation with light restores the high affinity to eDHFR. This strategy had previously been applied in the SLIPT system.^38,39^ Thus, we aimed at generating a **dual SLIPT** variant, named **dual SLIPT**^**NVOC**^ (**5**_**B**_), that allows for precise dimerization in both space and time (Figure 4). Here, nitroveratryl-based photocaging was chosen for its compatibility with green fluorescent protein during fluorescence imaging.

In contrast to constitutively active **dual SLIPT** without a photocaging group, incubation of cells expressing POIs fused to ^iK6^eDHFR and HOB with 10 μM **dual SLIPT**^**NVOC**^ first induces HOB recruitment to the PM. Irradiation of a defined area with a 405 nm laser then cleaves the caging group off of the TMP headgroup, whereupon ^iK6^eDHFR tagged POIs are rapidly recruited to the PM (Figures 4A, B and S4A,B, SI Movie 1). Translocation of the respective fluorescent proteins was evaluated by determining the ratio of signal intensity at the PM to signal intensity in the cytoplasm over time, and mapping that ratio to zero (minimal intensity) and one (maximal intensity) for both HOB-mScar- and ^iK6^eDHFR-mEGFP, respectively (Figure 4B). Due to photobleaching post-irradiation, the PM to cytoplasm signal intensity ratio was normalized to the average signal intensity ratio post-uncaging. **Dual SLIPT**^**NVOC**^ proved to be highly cell-permeant. Half-maximal HOB-mScar translocation occurred approximately 59 min post addition of **5**_**B**_ (IC50 95% CI: 52.66 to 65.17 min; *R*^2^ = 0.9074) (Figure S4C). Half-maximal dimerization (^iK6^eDHFR-mEGFP translocation) occurred 19s postirradiation (10s) with 405 nm (IC50 95% CI: 0.2310 to 0.3988 min; *R*^2^ = 0.6489) (Figure S4C). As uncaged **dual SLIPT**^**NVOC**^ is identical to **dual SLIPT**, we assumed that dual recruitment induced by either lipid probe is equally capable of dimerizing and that the primary difference lies in recruitment kinetics and spatial precision.

### Using Photoactivatable **Dual SLIPT^NVOC^** to Spatiotemporally Control Lamellipodia

After verification of **dual SLIPT**^**NVOC**^ functionality, we now wished to utilize this tool to elicit a functional response by controlling protein activity through two subsequent cytoplasm-to-PM recruitment events, as well as the subcellular dimerization of the recruited two POIs. As lamellipodia formation is a well-understood output, we chose this cellular event to generate a proof-of-principle that **dual SLIPT**^**NVOC**^ can be used to verify signaling hierarchies and control signaling outcomes. In principle, though, other signaling events where PM-recruitment is predicted to control protein function, such as pleckstrin homology (PH)-^40^ or phox (PX)-domain^41^ containing, or transmembrane receptor-associated,^42^ as well as lipidated^43^ proteins, could be investigated.

Lamellipodia are protrusions of the PM caused primarily by Rac1, a member of the Rho GTPases, inducing network-like polymerization of actin.^44,45^ Rac1, itself, is regulated by a large number of guanine nucleotide exchange factors (GEFs) that are activatory, and deactivatory GTPase activating proteins (GAPs).^46,47^ Lipid-mediated recruitment to the PM is a primary regulatory mechanism of GEF^48^ and GAP^47^ function. In the case of the prototypical Rac1-specific GEF, T-cell lymphoma invasion and metastasis 1 (Tiam1),^49,50^ and GAP, breakpoint cluster region (BCR),^51,52^ this recruitment event and resulting GEF/GAP complex formation at the PM has been shown to modulate Rac1 activity in processes such as synaptogenesis.^53,54^ Therein, Tiam1-activated Rac1 is subsequently deactivated by the BCR–Tiam1 interaction.

To demonstrate **dual SLIPT**^**NVOC**^ use in validating this signaling sequence, we serum-starved NIH 3T3 cells, expressing ^iK6^eDHFR-mEGFP-BCR^Rho GAP^ and HOB-mScarlet-Tiam1^DH/PH^, as well as staining F-actin with 500 nM SiR-XActin^55^ overnight. POI localization was assessed by the visualization of the respective fluorescent protein using confocal imaging, before incubation with 5 μM of **dual SLIPT**^**NVOC**^ (**5**_**B**_) in DMEM(−).

**Figure 5:**
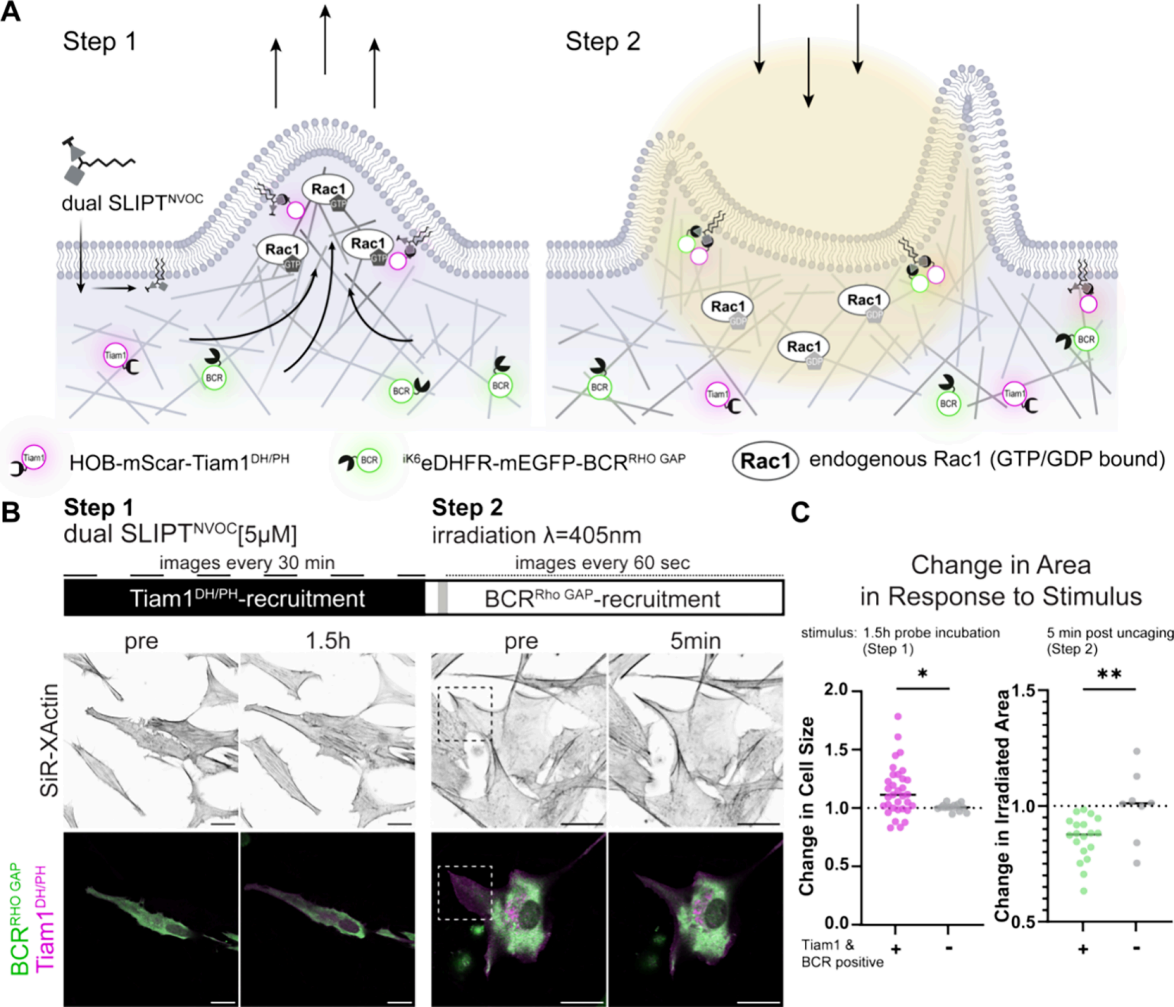
**Dual SLIPT^NVOC^** enables synthetic control over lamellipodial signaling with subcellular resolution. (A) Schematic depicting the expected cyto-to-pm recruitment events, as well as their stepwise effect on endogenous Rac1. Incubation dual SLIPT^NVOC^ initially recruits the HOB-tagged DH/PH domain of Tiam1, which leads to whole cell activation of Rac1 and expansion of the cell. Thereafter, irradiation with 405 nm uncages the TMP headgroup with high spatiotemporal precision, which rapidly induces corecruitment of the ^iK6^eDHFR-tagged Rho GAP domain of BCR, leading to retraction of the protrusion. (B) Imaging scheme and representative live cell images of cells expressing both HOB-mScar-Tiam1^DH/PH^ and ^iK6^eDHFR-mEGFP-BCR^Rho GAP^. Actin stained with 500 nM SiR-XActin. Images were acquired after overnight serum-starvation and simultaneous incubation with SiR-XActin. Pre (left) depicts cells preincubation of 5 μM dual SLIPT^NVOC^, pre (right) depicts cells preirradiation (dashed square). Scale bars 10 μm. (C) Quantitation of the response in Tiam1 and BCR positive cells (+; magenta and green dots), or cells expressing ^iK6^eDHFR-mEGFP and HOB-mScar (−, gray dots) to dual SLIPT^NVOC^ incubation for 1.5h (left), or subsequent subcellular irradiation (right) (post incubation: Tiam1 & BCR positive cells, *n* = 33; Tiam1 & BCR negative cells, *n* = 15), (*p* = 0.0132, Mann–Whitney test) (post irradiation: Tiam1 & BCR positive cells, *n* = 20; Tiam1 & BCR negative cells, *n* = 8), (*p* = 0.0081, Mann–Whitney test).

We hypothesized that this treatment would result in the following two-step scenario: In an initial step (step 1), **dual SLIPT**^**NVOC**^ appears at the inner leaflet of the PM, where it recruits HOB-tagged Tiam1^DH/PH^. This leads to PM-localized activation of endogenous Rac1 (Figure 5A). In the second step, irradiation of the PM-localized probe–protein complex causes uncaging of the NVOC-caged TMP moiety and rapid corecruitment of the ^iK6^eDHFR-tagged BCR^Rho GAP^, as well as subsequent deactivation of endogenous Rac1. As the GEF/GAP complex formation is based on the interaction of BCR with the region surrounding the N-terminal PH domain of Tiam1,^53^ both BCR and Tiam1 were truncated such that this interaction was suppressed in the constructs introduced (Figure S5K) without affecting their Rac1-modulating function.^56,52^ Therefore, dimerization of the two POIs is re-established only after incubation and irradiation of **dual SLIPT**^**NVOC**^. Cells expressing the same constructs, but lacking the respective GEF or GAP, were used as negative controls.

Using cell size as a proxy for Rac1 activity in step 1, we indeed found that after 1.5 and 3 h of incubation with 5 μM **dual SLIPT**^**NVOC**^, cells expressing HOB-mScar-Tiam1^DH/PH^ increased in cell size compared to their preincubation size (Figures 5B and S5A,C), as well as compared to cells expressing HOB-mScar, yet lacking exogenous Tiam1^DH/PH^ (*p* = 0.0132) (Figures 5C and S5E). Cell size did not meaningfully increase beyond 1.5 h post-incubation (Figure S5B), with median cell size changes after 1.5 h being 82.5 μm^2^. Cells expressing HOB-mScar, only, did not meaningfully respond with changes in cell size at any point measured (Figure S5D).

As **dual SLIPT**^**NVOC**^ was uncaged subcellularly in step 2, whole cell size was no longer an appropriate readout. Instead, lamellipodial retraction was assessed by changes in the irradiated area. Recruitment of ^iK6^eDHFR-mEGFP-BCR^Rho GAP^ caused meaningful retraction of protrusions (Figure 5B), indicative of successful GEF/GAP complex formation and downregulation of local endogenous Rac1 activity. Meanwhile, cells expressing ^iK6^eDHFR-mEGFP, but lacking BCR^Rho GAP^ did not show any significant change in the irradiated area (*p* = 0.6543) (Figure S5I). Median decrease of irradiated area caused by BCR^Rho GAP^ recruitment was to 87.5% (a median reduction of 23.3 μm^2^) of the preirradiation size (*p* < 0.0001) after just 5 min (Figure S5F–H), which was not meaningfully different between the 5- and the 10 min time points (*p* = 0.1232) (Figure S5G). Thus, the relative size after 5 min post irradiation in cells expressing BCR^Rho GAP^ was compared to irradiated cells, lacking the GAP (Figures 5C and S5J), which resulted in a significantly differing response (*p* = 0.0081).

In demonstrating the use of **dual SLIPT**^**NVOC**^ for verifying the underlying signaling hierarchy of a biological event, we can conclude that **dual SLIPT** represents the first self-localizing, PM-targeted (opto-)chemical system capable of dual recruitment and dimerization of any POIs fused to ^iK6^eDHFR and HOB.

## Summary and Conclusions

With **dual SLIPT**, we have generated SLIPT variants capable of dimerizing any two POIs, fused to ^iK6^eDHFR and HOB. **Dual SLIPT** is a two-tag-two-protein system that eliminates the need to prelocalize one of the partners to the PM or the use of an additional third protein tag. The constitutively active **dual SLIPT** affords parallel recruitment of the POIs, while **dual SLIPT**^**NVOC**^ enables light-controlled (conditional) dimerization, providing improved spatiotemporal control over the process. Thus, **dual SLIPT**^**NVOC**^ allows the investigation of recruitment events in which improved spatiotemporal control of dimerization is required, to clarify cause-and-effect relationships, which is often necessary in dynamic lipid-mediated signaling. This was demonstrated by controlling lamellipodia via stepwise recruitment of Tiam1, a Rac1-GEF, and BCR, a Rac1-GAP.

Using FRET, we demonstrated **dual SLIPT**’s ability to dimerize, rather than merely colocalize at the PM. This makes **dual SLIPT** optimal for interrogating cytosol-to-PM recruitment events at low, physiologically meaningful probe concentrations. Finally, due to its modular nature, the concept of **dual SLIPT** can easily be expanded to other cellular loci or used with other headgroup–protein tag pairs.

## Methods

### Synthesis

Detailed procedures for the synthesis of all compounds and their characterization, as well as all methods are available in the Supporting Information.

## Supporting Information Available

Supplementary figures and movie captions, synthesis and characterization of compounds, and supplementary methods for biological experiments (PDF)
**Dual SLIPT**^**NVOC**^-induced sequential dimerization of ^iK6^eDHFR-mEGFP and HOB-mScar in HeLa cells (AVI)

## Supplementary Material

cb4c00856_si_001.pdf

cb4c00856_si_002.avi
