# Supplementary material for: Dual SLIPT–A Lipid Mimic to Enable Spatiotemporally Defined, Sequential Protein Dimerization
Source: ACS Chem Biol. 2025 Apr 15;20(5):1038–47. doi: 10.1021/acschembio.4c00856 (PMC12090181; doi:10.1021/acschembio.4c00856)
Supplement: Supplementary file 1 — cb4c00856_si_001.pdf [file cb4c00856_si_001.pdf]

Supporting Information

Dual SLIPT – a lipid mimic to enable spatiotemporally defined, sequential protein dimerization

Kristina V. Bayer[1,2], Maedeh Taeb[1], Birgit Koch[1], Shige H. Yoshimura[3,4,5], Richard Wombacher[1,*]

[1] Department of Chemical Biology, Max Planck Institute for Medical Research, Jahnstraße 29, 69120 Heidelberg, Germany

[2] Heidelberg Biosciences International Graduate School (HBIGS), Heidelberg University, Im Neuenheimer Feld 501, 69120 Heidelberg, Germany

[3] Graduate School of Biostudies, Kyoto University, Kyoto 606-8501, Japan.

[4] Center for Living Systems Information Science (CeLiSIS), Kyoto University, 606-8501, Japan

[5] Institute for Integrated Cell-Material Sciences (iCeMS), Kyoto University, 606-8501, Japan

*Corresponding author: wombacher@mr.mpg.de

Table of Contents

Supplementary Figures 1

Protein optimization from HT7 to HOB 1

Ligand optimization to dual SLIPT 2

FRET results und controls 3

dual SLIPT^{NVOC} characterization 5

Control of Cellular protrusions using dual SLIPT^{NVOC} – Controls 6

Supplementary Methods 8

Synthesis 10

Reagent abbreviations 10

Synthesis 11

Supplementary Movie 20

cDNA 21

Supplementary Sequences 22

Publication bibliography 32

Supplementary Figures

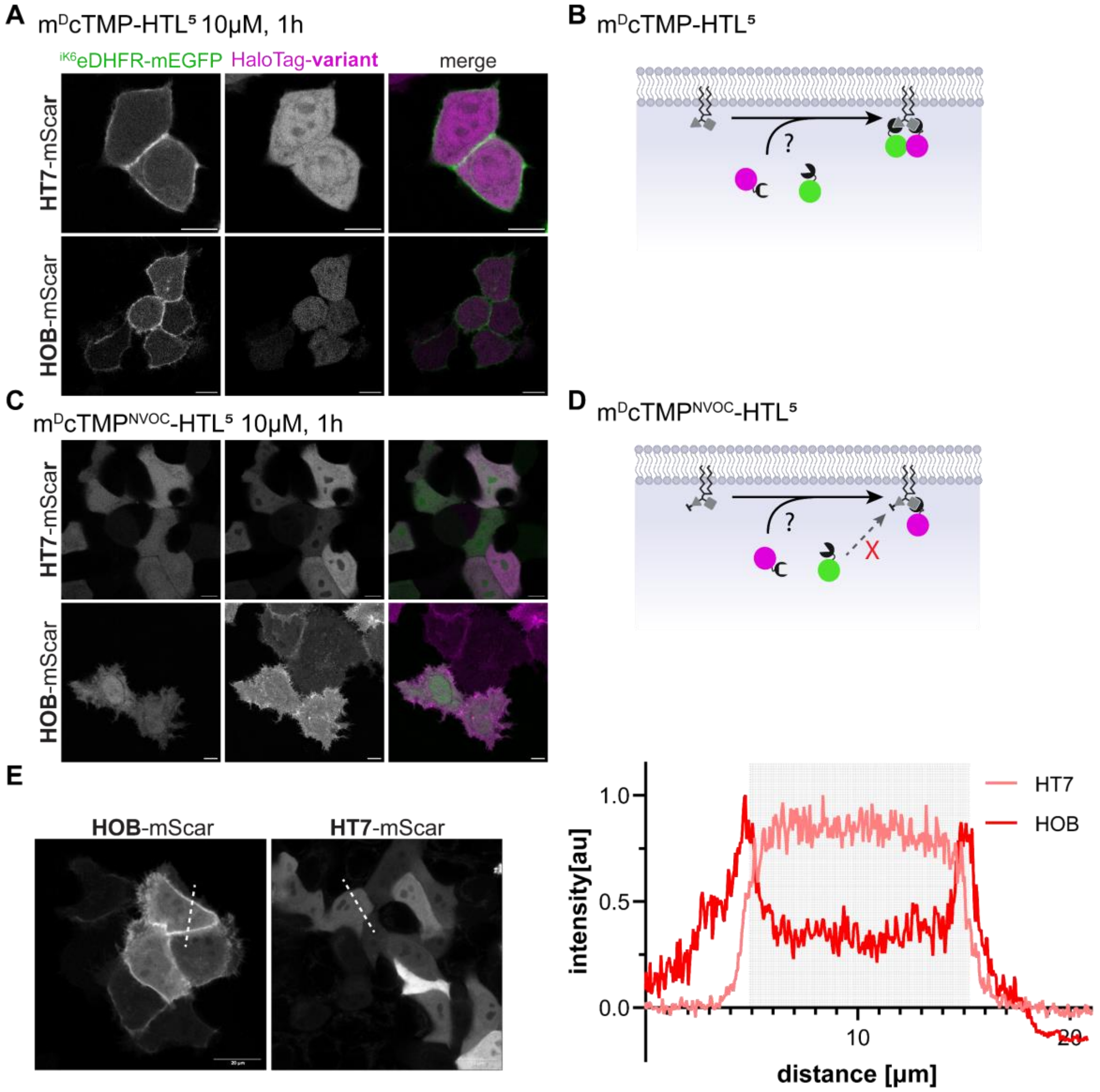

Figure S1 Protein optimization from HT7 to HOB.

A) Confocal fluorescence images of HeLa cells stably expressing HT7-mScar (top), or HOB-mScar (bottom), transfected with ^{iK6}eDHFR-mEGFP, and treated with 10 µM m^{D}cTMP-HTL^{5} (1A) for 1h before imaging. **B)** Corresponding schematic of translocation-read out: Cells, treated with m^{D}cTMP-HTL^{5} (1A) are assessed for translocation of both, ^{iK6}eDHFR-mEGFP, and the HT-variant used. **C)** Confocal fluorescence images of HeLa cells stably expressing HT7-mScar (top), or HOB-mScar (bottom), transfected with ^{iK6}eDHFR-mEGFP, and treated with 10 µM m^{D}cTMP^{NVOC}-HTL^{5} (1B) for 1h before imaging. **D)** Corresponding schematic of interrogated translocation-read out: As presence of the NVOC-protection group prevents ^{iK6}eDHFR-mEGFP translocation, cells, treated with m^{D}cTMP^{NVOC}-HTL^{5} (1B) are assessed only for translocation of the HT-variant used. **E)** Confocal images of HeLa cells expressing HOB-mScar, or HT7, treated with 10 µM m^{D}cTMP-HTL^{5} (1A) for 1h, as well as the corresponding fluorescence intensity cross-sections. Scale bar, 10 µm, unless otherwise indicated.

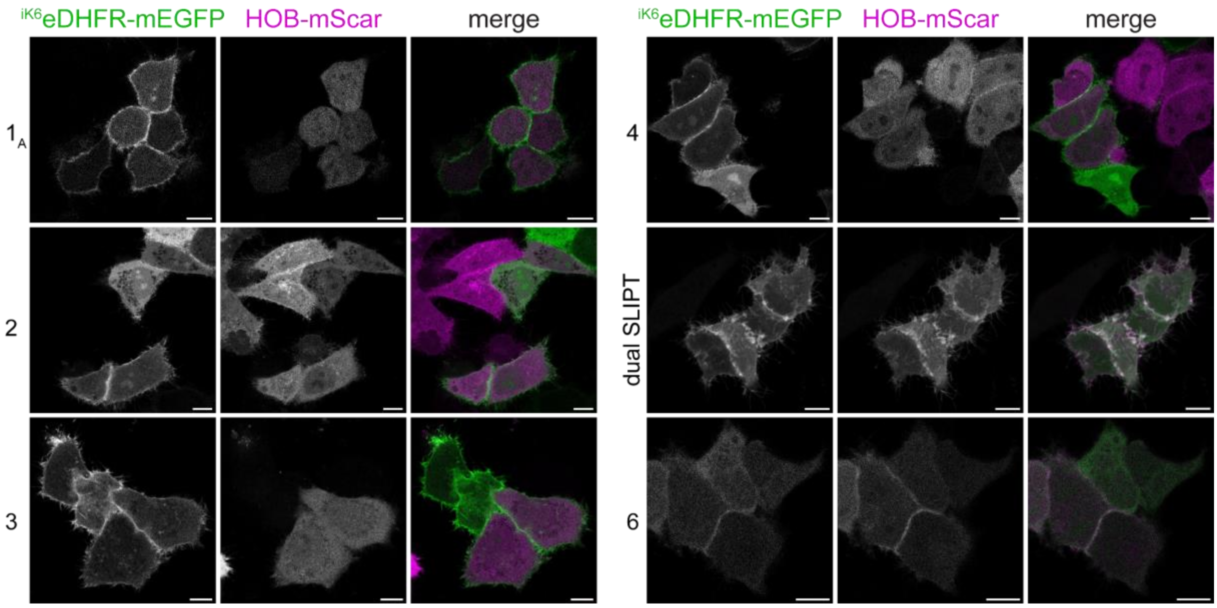

Figure S2 Ligand optimization from minimal ligand m^{D}cTMP-HTL^{5} (1A) to dual SLIPT (5A).
Confocal fluorescence images of HeLa cells stably expressing HOB-mScar, as well as ^{iK6}eDHFR-mEGFP, treated with 10 µM m^{D}cTMP-HTL^{5} (1A), m^{D}cTMP-HTL^{11} (2), m^{D}cTMP-HTL^{14} (3), m^{D}cTMP-HTL^{18} (4), dual SLIPT (5A), m^{D}cTMP-HTL^{21} (6), for overnight. Scale bar, 10 µm.

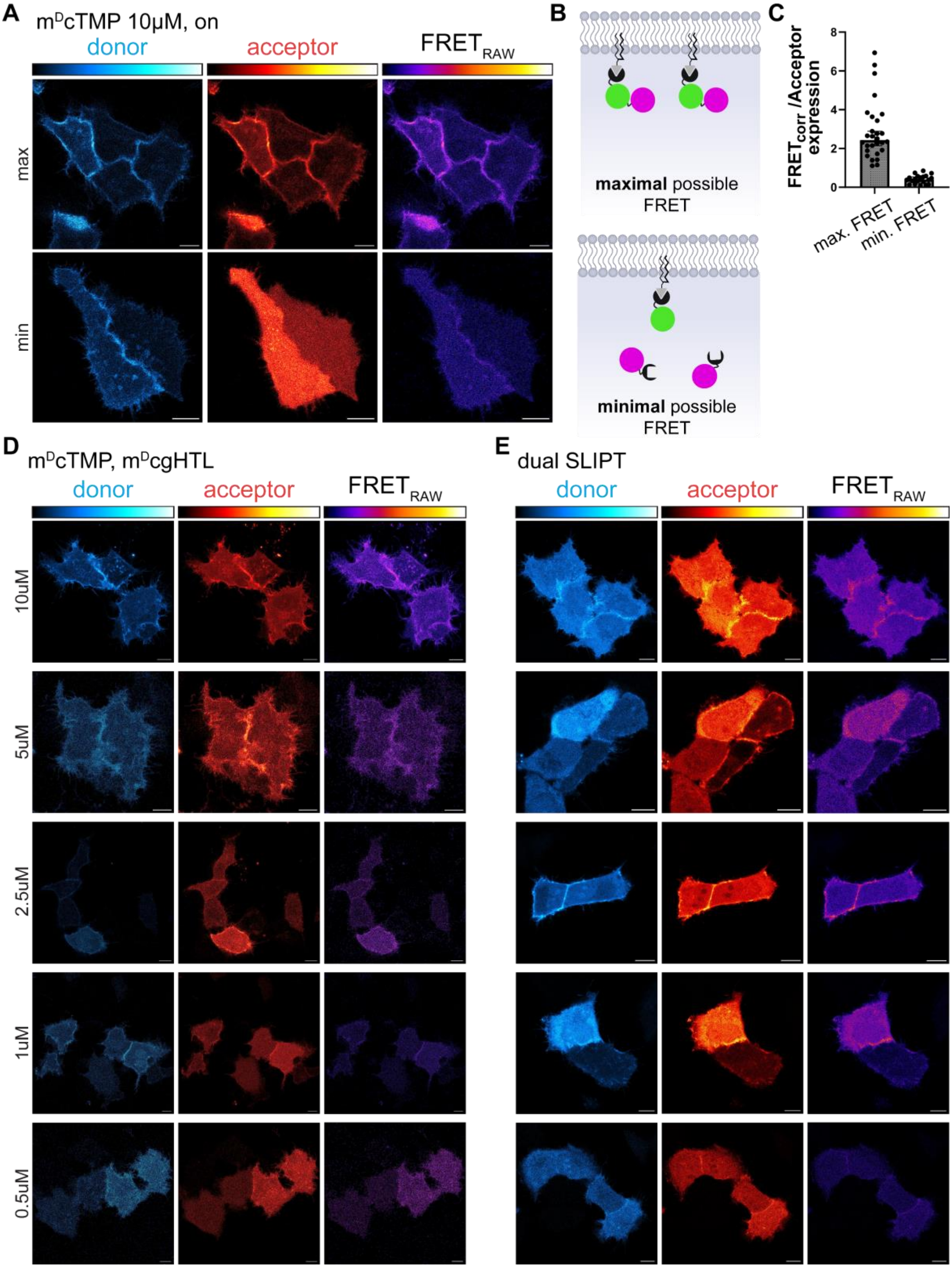

Figure S3 Minimal and maximal possible FRET, as well as raw data of FRET efficiency.

A) Confocal fluorescence images of HeLa cells expressing ^{iK6}eDHFR-mNeonGreen-mScar-I (top), and ^{iK6}eDHFR-mNeonGreen and HOB-mScar-I (bottom). Channels display donor signal excitation and detection in donor channel (Cyan hot), acceptor excitation and detection in acceptor channel (Red hot), and raw FRET signal intensity (Fire) Maximally possible FRET is assessed by recruiting the tandem construct ^{iK6}eDHFR-mNeonGreen-mScar-I to the PM, by incubating the cells with 10 µM m^{D}cTMP (7) for 30min pre-visualization (as indicated in the corresponding sketch in B). The minimal FRET condition was achieved by incubating the cells with 10 µM m^{D}cTMP (7) for 30min pre-visualization, which exclusively recruited

^{iK6}eDHFR-mNeonGreen to the PM (as indicated in the corresponding sketch in B). **B)** Schematic, displaying minimal and maximal possible FRET conditions. **C)** Quantification of the donor bleed-through, acceptor cross-excitation and acceptor intensity corrected FRET efficiency in the maximal/minimal possible FRET conditions. The difference between maximal (n = 28 fields of view, >30000 ROIs) and minimal (n = 20 fields of view, >15000 ROIs) $FRET_{CORR}$ was determined to be 6.05-fold ($p < 0.0001$, Mann-Whitney test). **D)** Representative confocal images of HeLa cells expressing ^{iK6}eDHFR-mNeonGreen and HOB-mScar-I, incubated with a mixture of m^{D}cTMP (7), and m^{D}cgHTL (8), 0.5-10 µM, each. **E)** Representative confocal images of HeLa cells expressing ^{iK6}eDHFR-mNeonGreen and HOB-mScar-I, incubated with dual SLIPT (5A) 0.5-10 µM. Scale bar, 10 µm.

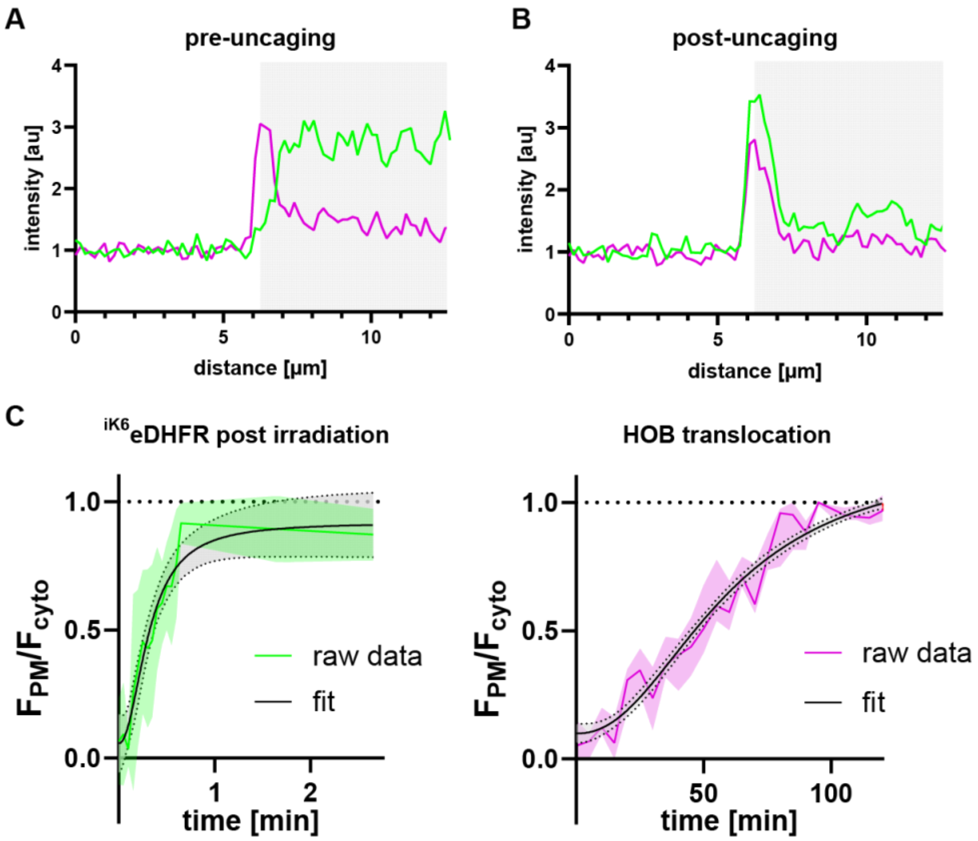

Figure S4 Response of iK6eDHFR-mEGFP, and HOB-mScar to incubation and subsequent irradiation of **dual SLIPT^{NVOC} (5B)**.

A) & **B)** Representative fluorescence intensity cross-sections of iK6eDHFR-mEGFP, and HOB-mScar pre- vs post-local uncaging of dual SLIPT^{NVOC} (5B). Signal is normalized to average extracellular fluorescence intensity. Grey shading indicates the area occupied by a cell. **C)** Ratio of plasma membrane localized to cytosolic fluorescence intensity of HOB-mScar (magenta, right) in response to incubation with 10 µM dual SLIPT^{NVOC} (5B), or of iK6eDHFR-mEGFP (green, left) in response to uncaging of dual SLIPT^{NVOC} (5B) over time. Raw data is fitted with sigmoidal functions. HOB: Hill Slope: 2.215; $t_{1/2}$: 58.91 min (95% CI 52.66 to 65.17); R^2: 0.9074; iK6eDHFR: Hill Slope: 2.114; $t_{1/2}$: 0.3149 min (95% CI 0.2310 to 0.3988 min); R^2: 0.6489.

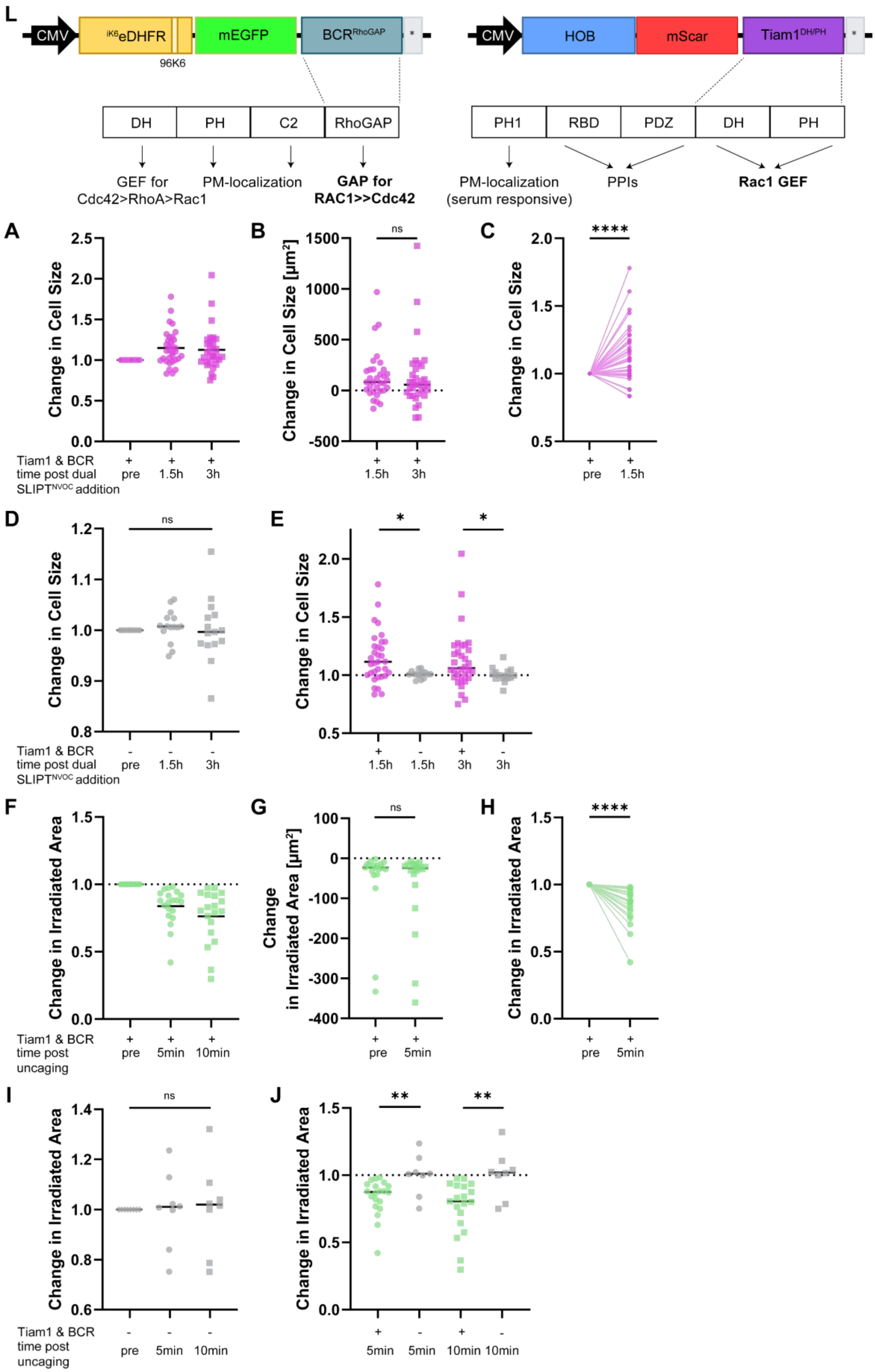
L
CMV
iK6eDHFR
96K6
mEGFP
BCRRhoGAP
CMV
HOB
mScar
Tiam1DH/PH
DH
PH
C2
RhoGAP
GEF for Cdc42>RhoA>Rac1
PM-localization
GAP for RAC1>>Cdc42
PH1
RBD
PDZ
DH
PH
PM-localization (serum responsive)
PPIs
Rac1 GEF
A
B
C
Change in Cell Size
Change in Cell Size [µm²]
Tiam1 & BCR
time post dual SLIPTNVOC addition
pre
1.5h
3h
ns
****
D
E
*
Change in Irradiated Area
Change in Irradiated Area [µm²]
F
G
H
time post uncaging
5min
10min
I
J
**

Figure S5 dual SLIPT^{NVOC} (5B) enables synthetic control over lamellipodial signaling with subcellular resolution – extended data.

A) Quantitation of the change in cell size in response to dual SLIPT^{NVOC} incubation and consequent Tiam1 recruitment in cells expressing exogenous Tiam1$^{DH/PH}$ and BCR$^{Rho\ GAP}$, normalized to pre-incubation size. After 1.5h: mean cell size was 1.15 times, and after 3h mean size was 1.13 times the pre-incubation size, (n > 30 cells per condition). **B)** Difference in cell size (absolute numbers) in response to dual SLIPT^{NVOC} incubation and consequent Tiam1 recruitment in cells expressing exogenous Tiam1$^{DH/PH}$ and BCR$^{Rho\ GAP}$. Median cell size changes after 1.5h was 82.5 µm^2, while cell size after 3h was increased by a median value of 57.4 µm^2 (p = 0.5722; Wilcoxon test [paired, nonparametric]). **C)** Quantitation of the change in cell size in response to dual SLIPT^{NVOC} incubation and consequent Tiam1 recruitment in cells expressing exogenous Tiam1$^{DH/PH}$ and BCR$^{Rho\ GAP}$, normalized to pre-incubation size. After 1.5h: mean cell size was 1.15 times (p <0.0001; Kolmogorov-Smirnov test [unpaired, nonparametric]), (n > 30 cells per condition). **D)** Quantitation of the change in cell size in response to dual SLIPT^{NVOC} incubation and consequent HOB-mScar recruitment in cells lacking exogenous Tiam1$^{DH/PH}$ and BCR$^{Rho\ GAP}$, normalized to pre-incubation size. After 1.5h: mean cell size was 1.007 times, and after 3h 0.9966 times the pre-incubation size (p = 0.2818; Friedmann ANOVA [matched, nonparametric]). **E)** Extension of figure 5C. Magenta: cells expressing exogenous Tiam1$^{DH/PH}$ and BCR$^{Rho\ GAP}$; grey: cells lacking exogenous Tiam1$^{DH/PH}$ and BCR$^{Rho\ GAP}$. Relative cell size after 1.5 h (p = 0.0132, Mann-Whitney test [unpaired, nonparametric]), or 3 h (p = 0.0357, Mann-Whitney test [unpaired, nonparametric]) in response to dual SLIPT^{NVOC} incubation. **F)** Quantitation of the change in size of irradiated area in response to subcellular dual SLIPT^{NVOC} irradiation and consequent BCR recruitment in cells expressing exogenous Tiam1$^{DH/PH}$ and BCR$^{Rho\ GAP}$, normalized to pre-irradiation size. After 5 min: mean area was 0.88 times, and after 10 min mean area was 0. 81 times the pre-irradiation size, (n = 20 cells per condition). **G)** Difference in irradiated area (absolute numbers) in response to dual SLIPT^{NVOC} irradiation and consequent BCR recruitment in cells expressing exogenous Tiam1$^{DH/PH}$ and BCR$^{Rho\ GAP}$. Median area changes after 5min was -23.3 µm^2, while area after 10 min was decreased by a median value of 24.7 µm^2 (p = 0.1232; Wilcoxon test [paired, nonparametric]). **H)** Quantitation of the change in size of irradiated area in response to dual SLIPT^{NVOC} irradiation and consequent BCR recruitment in cells expressing exogenous Tiam1$^{DH/PH}$ and BCR$^{Rho\ GAP}$, normalized to pre-irradiation size. After 5 min: mean cell size was 0.84 times (p <0.0001; Kolmogorov-Smirnov test [unpaired, nonparametric]), (n = 20 cells per condition). **I)** Quantitation of the change in size of irradiated area in response to dual SLIPT^{NVOC} irradiation and consequent ^{iK6}eDHFR-mEGFP recruitment in cells lacking exogenous Tiam1$^{DH/PH}$ and BCR$^{Rho\ GAP}$, normalized to pre-irradiation size. After 5 min: mean cell size was 1.01 times, and after 10 min 1.02 times the pre-irradiation size (p = 0.6543; Friedmann ANOVA [matched, nonparametric]). **J)** Extension of figure 5C. Green: cells expressing exogenous Tiam1$^{DH/PH}$ and BCR$^{Rho\ GAP}$; grey: cells lacking exogenous Tiam1$^{DH/PH}$ and BCR$^{Rho\ GAP}$. Relative size of irradiated area after 5 min (p = 0.0081, Mann-Whitney test [unpaired, nonparametric]), or 10 min (p = 0.0054, Mann-Whitney test [unpaired, nonparametric]) in response to subcellular dual SLIPT^{NVOC} irradiation. K) Schematic of full length BCR (left) and Tiam1 (right) domains, as well as crude annotation of domain function. ^{iK6}eDHFR-mEGFP-BCR$^{Rho\ GAP}$ contained only the Rac1 specific RhoGAP domain of BCR; HOB-mScar- Tiam1$^{DH/PH}$ contained only the DH and PH domains of full length Tiam1.

Supplementary Methods

Plasmid Construction.

Both cDNA and peptide sequences can be found listed in the Supporting Information (Supplementary sequences). As plasmid backbone, we used pcDNA5/FRT (purchased from Thermo Fisher Scientific). To construct ^{iK6}eDHFR-mEGFP, eDHFR was first fused to meGFP, before introducing the hexa-lysine sequence (iK6) between Asp69 and Asp70 in eDHFR by Site-directed Mutagenesis (Q5® Site-Directed Mutagenesis Kit, New England Biolabs). mScarlet-I was generated from mScarlet by mutation of Thr74 to Ile, using PrimeStar Max (Takara Bio). ^{iK6}eDHFR-mNG-P2A-HOB-mScarI was constructed by first fusing the protein-tags to their respective fluorescent proteins, before introducing a P2A site using Gibson cloning with the reverse primer covering the entire insertion site. The SGLSKGEE linker between mNeonGreen and mScarlet-I in ^{iK6}eDHFR-mNG-mScarI, as optimized (McCullock et al. 2020), was constructed the same way, post-fusion of ^{iK6}eDHFR-mNG and mScarlet-I. HOB-mScar-Tiam1$^{DH/PH}$ and ^{iK6}eDHFR-mEGFP-BCR$^{Rho\ GAP}$ were generated by subcloning of the respective domains into HOB-mScar and ^{iK6}eDHFR-mEGFP. Linker regions were generated using long oligos (eurofins). ^{iK6}eDHFR-mEGFP, HOB-Scar, ^{iK6}eDHFR-mNG-mScarI, ^{iK6}eDHFR-mNG-P2A-HOB-mScarI and ^{iK6}eDHFR-mEGFP-BCR$^{Rho\ GAP}$, and HOB-mScar-Tiam1$^{DH/PH}$ were generated using standard Gibson cloning, while ^{iK6}eDHFR-mNG was constructed using In-Fusion cloning (Takara Bio).

Cell Culture and Transfection.

HeLa (RRID:CVCL_0030) and HeLa Kyoto Flp-In™ cells, gifted by Dr. Amparo Andres-Pons (EMBL, Heidelberg), were cultured in high glucose DMEM (Sigma Aldrich), which was supplemented with 2 mM L-glutamine, 1 mM sodium pyruvate, and 10% heat-inactivated sterile-filtered FBS [DMEM(+)], at 37 °C under a 5% CO_2 atmosphere. 3T3 Flp-In™ cells (RRID:CVCL_U422; Thermo Fisher Scientific) were cultured in high glucose DMEM (Sigma Aldrich), which was supplemented with 2 mM L-glutamine, 1 mM sodium pyruvate, and 10% NCS (new born calf serum). For transient expression experiments, cells were transfected using Lipofectamine3000 (Thermo Fisher Scientific) in accordance with the manufacturer's instructions.

Stable Cell lines.

Stable incorporation of HOB-mScar between the FRT-sites was conducted by co-transfecting HeLa Kyoto Flp-In™ with pOG44 (invitrogen) and the pcDNA5/FRT-HOB-mScar plasmid in a 9:1 (w/w) ratio in DMEM(+) for 2 days, before adding and selecting for stably expressing cells with Hygromycin [100µg/mL] for at least 3 days. Single clone sorts were used.

Microscopy.

Confocal fluorescence imaging was performed on a SP8 microscope (Leica), equipped with a White Light Laser and an incubation chamber (The Box, Life Imaging Services). Images were acquired with either a HC PL APO CS2 43x/1.1NA water objective, or a HC PL APO CS2 63x/1.4NA oil objective. Photo-uncaging was performed for 10s with a 405nm diode laser with a power of 0.26 mW for defined regions of interest (ROI). Settings for confocal fluorescence imaging were as follows: mEGFP 488 nm ex., 494 nm – 556 nm em.; mNeonGreen 505 nm ex. 515-549nm em., mScarlet and mScarlet-I 569 nm ex. 579-694 nm em.. Unless otherwhise stated, images were captured at 37°C, staggered. Addition of dual SLIPT derivatives to cells [0.1 - 20 µM] and SiR-XActin [500 nM] was conducted in FBS- and phenolred-free high glucose medium [DMEM(-)]. The final DMSO fraction did not exceed 1% (v/v). Images were analyzed using Fiji (ImageJ).

SLIPT assay.

To verify compound functionality (ability to colocalize both POIs), HeLa cells stably expressing HOB-mScar (pcDNA5/FRT-HOB-mScarlet) were seeded into multi-well microscopy dishes (µ-Slide 18 well glass bottom, Ibidi, $5x10^4$ cells/well), and cultured for 16h. Thereafter, they were transfected with

^{iK6}eDHFR-mEGFP (pcDNA5/FRT- ^{iK6}eDHFR-mEGFP) for 6h, before incubation with 10 μM of compound 1-5 in DMEM(-) o/n.

For time-resolved imaging of compound-induced, sequential recruitment of ^{iK6}eDHFR-mEGFP and HOB-mScar, 5x10^{4} cells/well were seeded into μ-Slide VI 0.4 IbiTreat (Ibidi). Transfection occurred after 24h, for 8h, before changing medium to DMEM(-) and observing under the microscope. Thereupon, 10 μM of dual SLIPT^{NVOC} was added, and images acquired every 5 minutes for 120 minutes, before uncaging dual SLIPT^{NVOC} with 405 nm, and imaging every 5 seconds.

FRET-assay.

Wavelengths used for donor excitation: 505nm; 569nm for mScarlet-I. Detector settings used: 515-549 nm (donor); 579-694 nm (acceptor). Images were obtained in the following sequence: donor-only excitation, and simultaneous detection of donor and acceptor ($FRET_{RAW}$) channels; thereafter acceptor-only excitation and simultaneous detection of donor and acceptor channels.

To test the ability for one molecule of compounds 1-5 to simultaneously bind both ^{iK6}eDHFR- and HOB-tagged POIs, 5x10^{4} HeLa Kyoto cells/well were seeded onto multi-well microscopy dishes (18 well glassbottom μ-Slides (Ibidi) and cultured for 16h. Thereafter, they were transfected with (i) ^{iK6}eDHFR-mNG (pcDNA5/FRT- ^{iK6}eDHFR-mNeonGreen), or (ii) HOB-mScar-I (pcDNA5/FRT-HOB-mScarlet-I) as single expression controls, or (i) ^{iK6}eDHFR-mNG-mScarI (pcDNA5/FRT-^{iK6}eDHFR-mNG-mScarlet-I) as positive control, or (iii) ^{iK6}eDHFR-mNG-P2A-HOB-mScarI (pcDNA5/FRT-^{iK6}eDHFR-mNG-P2A-HOB-mScarlet-I) for 6h, before incubation with m^{D}cTMP (i), m^{D}cHTL (ii) alone, or combined (iii), or with dual SLIPT (iii) in DMEM(-) o/n [0.5-20 μM].

Masks based on ROIs were determined by donor (i), or acceptor localization (ii), (iii). To do so, the respective images were thresholded for 2% highest pixel intensity, which filtered out cytosolic and extracellular fluorescent signal. The resulting masks were superimposed with the image to be analyzed and only the regions included by the masks were measured. $FRET_{RAW}$ was corrected for acceptor cross-excitation (0.0781± 0.0160; 0.4030± 0.0693; 0.0632± 0.0078) and donor bleed-through (0.1233± 0.0080; 0.3237± 0.0435; 0.1304± 0.0039) (25% highest intensity pixels), (Spiering et al. 2013) by multiplying average values determined by single expression controls (n=8 per biological replicate) with the $FRET_{RAW}$ images in Fiji, before normalizing the resulting $FRET_{CORR}$ value by acceptor intensity for each cell (n > 8k ROIs for each condition). $FRET_{CORR}$ values exceeding, or falling short of maximal, or minimal physically possible $FRET_{CORR}$, as determined by positive (recruitment of tandem construct) and negative control (recruitment of only donor) were excluded from quantitation.

Lamellipodial Control.

To synthetically control lamellipodial protrusions 3T3 Flp-In^{TM} were seeded into multi-well microscopy dishes (μ-Slide 18 well glass bottom, Ibidi, 1x10^{4} cells/well), and cultured for 16h. Thereafter, they were transfected with ^{iK6}eDHFR-mEGFP-BCR$^{Rho\ GAP}$ (pcDNA5/FRT- ^{iK6}eDHFR-mEGFP-BCR$^{Rho\ GAP}$), and HOB-mScar-Tiam1$^{DH/PH}$ (pcDNA5/FRT- HOB-mScar-Tiam1$^{DH/PH}$), using 150ng/well DNA total (1:2 ratio), or with ^{iK6}eDHFR-mEGFP (pcDNA5/FRT- ^{iK6}eDHFR-mEGFP), and HOB-mScar (pcDNA5/FRT- HOB-mScar), using 100ng/well DNA total (1:1 ratio) for 6h. Thereafter cells were washed with phenolred-free DMEM(-), before incubation with 500 nM SiR-XActin in DMEM(-) o/n. Cells were imaged without changing medium and dual SLIPT^{NVOC} was added to the wells to a final concentration of 5 μM. Images were acquired every 30 minutes for 180 minutes, before uncaging dual SLIPT^{NVOC} with 405 nm, and imaging every minute for 10 minutes.

Synthesis

General materials and methods.

All chemical reagents and solvents were purchased from commercial suppliers (Acros, Merck KGaA, Roth, Sigma-Aldrich, TCI) and used without further purification. Water-free solvents were stored over molecular sieves and used directly from a sealed-bottle. Thin layer chromatography (TLC) was performed on POLYGRAM® SIL G/UV_{254} precoated aluminium sheets (Macherey-Nagel) and visualized by fluorescence quenching. Flash column chromatography was performed using Biotage® Sfär DLV empty columns with the Isolera™ System (Biotage). Reversed-phase HPLC was performed either on a Thermo Scientific UltiMate300 UHPLC system with UV detection at 260 nm using a Supelco Analytical C18 column (25 × 212nm), or on a Shimadzu UltiMate300 UHPLC system with MS detection using a Shimadzu Shim-pack GIS preparative C18 column (50 × 250nm) or HiChrom Vydac 214TP 10µm C4 column (22 × 250mm). 1H NMR spectra were recorded on 298 K on a BRUKER Advance III HD 400 NMR spectrometer equipped with a CryoProbe™ (^{1}H: 400 MHz, ^{13}C: 101 MHz), and analysed via MestReNova 14.1.0. High-resolution mass spectra were acquired on a maXis II™ ETD-HRMS system (Bruker) using electron spray ionization (ESI).

Reagent abbreviations

DCM: Dichloromethane
DIC: N,N-diisopropylcarbodiimide
DIPEA: N,N-diisopropylethylamine
DMAP: 4-dimethylaminopyridine
DMF: N,N-dimethylformamide
Fmoc: Fluorenylmethyloxycarbonyl
HATU: O-(7-azabenzotriazol-1-yl)-N,N,N′,N′-tetramethyluronium-hexafluorphosphate
HOBt: 1-hydroxybenzotriazole (monohydrate)
MeOH: Methanol
PyBOP: benzotriazol-1-yloxytripyrrolidinophosphonium hexafluorophosphate
TFA: trifluoroacetic acid
TIS: triisopropylsilane

Synthesis

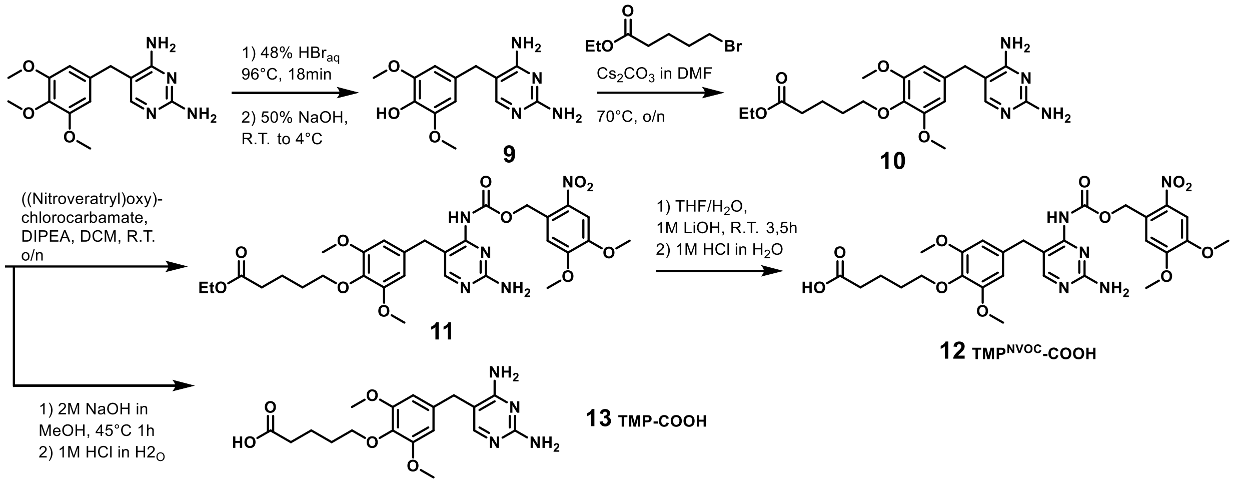

Synthesis of (9)

Compound 9 was synthesized according to (Ando et al. 2007). Trimethoprim (6g, 20.7mmol, 1 eq.) was added into 48% HBr (48%, 80 mL), which had been heated to 95°C. The reaction was stirred at 95°C for 18 min, while the progression of the reaction was continuously monitored using LCMS. The mixture was cooled to 0°C using an ice bath, after which sodium hydroxide (2M, 50 mL) was added via syringe. The flask was placed at 4 °C overnight. The precipitate was filtered and washed with ice-cold H_2O. Recrystallization was performed in boiling H_2O. The mixture was neutralized using 2N NaOH aq. (~ pH 7.0), and again placed at 4°C. The product was washed with H_2O, and dried under vacuum (2.94g, 10.7mmol, 55%) to afford white crystals.

^{1}H NMR (400 MHz, *d*-DMSO): δ 7.37 (s, 1H), 6.79 (brs, 2H), 6.49 (s, 1H), 6.30 (brs, 2H), 5.68 (s, 2H), 2.86 (s, 6H), 2.67 (s, 2H). brs = broad singlet.
HRMS (ESI): calculated for $[M+H]^+$, 277.1295; found, 277.1298.

Synthesis of (10)

Synthetic procedure for **compound 10** was adapted from (Ballister et al. 2014). In a flame-dried flask, **9** (1.00g, 3.62mmol, 1.00 eq.) was dissolved in anhydrous DMF (5 mL). Caesium carbonate (2.36g, 7.24 mmol, 2.00 eq.) was added, and the mixture was heated to 70°C. Then, Ethyl-5-bromovalerate (0.87 µL, 5.43 mmol, 1.50 eq.) was added and the reaction was stirred at 70°C for 16h. Thereafter, the mixture was filtered, concentrated under vacuum, and the crude product was purified by column chromatography on silica gel, using a gradient of 5-10% MeOH in DCM. **Compound 10** (1.02g, 2.52 mmol, 70%) was obtained as a brown solid. The spectroscopic data of **compound 10** are in agreement with the reported data.

^{1}H NMR (400 MHz, CD_3OD): δ 7.52 (s, 1H), 6.53 (s, 2H), 4.14 (q, *J* = 7.1 Hz, 2H), 3.92 (t, *J* = 6.0 Hz, 2H), 3.80 (s, 6H), 3.66 (s, 2H), 2.41 (t, *J* = 7.4 Hz, 3H), 1.88 – 1.78 (m, 2H), 1.78 – 1.66 (m, 2H), 1.26 (t, *J* = 7.1 Hz, 3H).
HRMS (ESI): calculated for $[M+H]^+$, 405.2132; found, 405.2131.

Synthesis of (11)

Compound 10 (500mg, 1.24 mmol, 1.00eq.) was dissolved in DCM (5mL), and DIPEA (204 µL, 1.24 mmol, 1.00 eq.) was added. The mixture was stirred at rt for 5 min, after which (Nitroveratryl)oxy)chlorocarbamate (341mg, 1.24 mmol, 1.00 eq.) was added. The mixture was stirred at rt in the dark for 16 h, before it was diluted with DCM (50mL). The solvent was evaporated, and the crude product purified over column chromatography on silica gel using a gradient of 0-10% MeOH in

DCM. **Compound 11** (83.0mg, 129 μmol, 22%) was separated from its regioisomer and obtained as a pale-yellow solid.

^{1}H NMR (400 MHz, CD_3OD): δ 8.00 (s, 1H), 7.76 (s, 1H), 7.14 (s, 1H), 6.46 (s, 2H), 5.53 (s, 2H), 4.13 (q, *J* = 7.1 Hz, 2H), 3.93 (s, 3H), 3.89 (s, 3H), 3.86 (t, *J* = 4.8 Hz, 2H), 3.83 (s, 2H), 3.73 (s, 6H), 2.40 (t, *J* = 7.4 Hz, 2H), 1.89 – 1.76 (m, 2H), 1.76 – 1.65 (m, 2H), 1.26 (t, *J* = 7.1 Hz, 3H).
HRMS (ESI): calculated for $[M+H]^+$, 644.2562; found, 644.2566.

Synthesis of TMP^{NVOC}-COOH (12)

Synthesis was adapted from (Yoshii et al. 2021). To a solution of **compound 11** (70mg, 109 μmol, 1.00 eq.) in THF (1.4mL) and water (1.4mL), 1M LiOH aq. (280 μL; 109 μmol, 1.00 eq.) was added. The reaction was stirred at rt in the dark for 4h. The reaction was diluted with water (15mL) and acidified with 1M HCl to pH 3. The crude product was extracted using Ethyl acetate (3 x 30mL) and the organic phase was dried via anhydrous $MgSO_4$. After filtration, the solvent was evaporated and the crude resuspended in DMSO, before it was purified via reverse-phase C18 HPLC, using a linear gradient of 20-70% ACN in H_2O (0.1%TFA). **Compound 12** (45.0mg, 73.1 μmol, 67%) was obtained as a pale-yellow solid.

^{1}H NMR (400 MHz, CD_3OD): δ 8.00 (s, 1H), 7.76 (s, 1H), 7.14 (s, 1H), 6.46 (s, 2H), 5.53 (s, 2H), 4.13 (q, *J* = 7.1 Hz, 2H), 3.93 (s, 3H), 3.89 (s, 3H), 3.86 (t, *J* = 4.8 Hz, 2H), 3.83 (s, 2H), 3.73 (s, 6H), 2.40 (t, *J* = 7.4 Hz, 2H), 1.89 – 1.76 (m, 2H), 1.76 – 1.65 (m, 2H), 1.26 (t, *J* = 7.1 Hz, 3H).
HRMS (ESI): calculated for $[M+H]^+$, 616.2249; found, 616.2257

Synthesis of TMP-COOH (13)

To a solution of **compound 10** (200mg, 0.45 mmol, 1.00 eq.) in MeOH (2mL), 2M NaOH aq. (0.74 mL, 1.48 mmol, 3.00 eq.) was added. The mixture was stirred at rt for 1h before evaporating the solvent. The crude was re-dissolved in water (10 mL) and then brought to pH 4 with 1 M HCl aq.. Fine, white precipitate formed, which was and washed with ice-cold water to afford **compound 13** (60mg, 0.16mmol, 30%) as a white solid.

^{1}H NMR (400 MHz, CD_3OD): δ 7.32 (s, 1H), 6.52 (s, 2H), 3.94 (t, *J* = 5.9 Hz, 2H), 3.79 (s, 6H), 3.64 (s, 2H), 2.27 (t, *J* = 7.1 Hz, 2H), 1.80 – 1.66 (m, 4H).
HRMS (ESI): calculated for $[M+H]^+$, 377.1819; found, 377.1823.

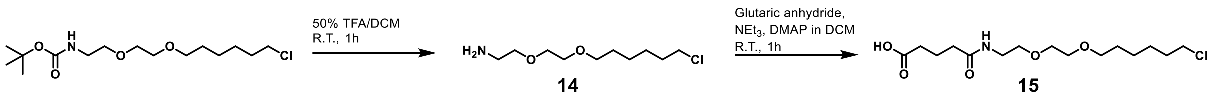

Synthesis of (15)

Boc-protected HTL (100 mg, 307 μmol, 1 eq.) was dissolved in DCM (2ml), and stirred at rt, before TFA (1 ml, 15 mmol, 45 eq.) was added. The reaction was complete after 1h, the crude was diluted with 2ml water and extracted with Ethyl Acetate (3x 4ml). The organic fractions were combined and the solvent evaporated to render the oily product, HTL-amine **(14)**. Yield: quantitative.

^{1}H NMR (400 MHz, DMSO) δ 7.65 (s, 4H), 3.63 (t, *J* = 6.6 Hz, 2H), 3.60 – 3.54 (m, 4H), 3.53 – 3.48 (m, 2H), 3.38 (t, *J* = 6.6 Hz, 2H), 2.96 (t, *J* = 5.3 Hz, 2H), 1.76 – 1.65 (m, 2H), 1.49 (q, *J* = 7.0 Hz, 2H), 1.45 – 1.26 (m, 4H).
HRMS (ESI): calculated for $[M+H]^+$, 224.1412; found, 224.1406.

HTL-amine **(14)** (50 mg, 223 μmol, 1 eq.) was used without further purifications, by dissolving it in dry DCM (2.5 mL), adding triethylamine (155 μL, 1.12 mmol, 5 eq.), glutaric anhydride (25.5 mg, 223 μmol, 1 eq.) and 4-dimethylaminopyridine (24.6 mg, 201 μmol, 0.9 eq.). The reaction was stirred for 1h, before

addition of (18 mL) 1N HCl to neutralize. Ethyl acetate was used to extract the product **15** (yield: quant.), which was thereafter dried under reduced pressure.

^{1}H NMR (400 MHz, $CDCl_3$) δ 6.19 (s, 1H), 3.61 (s, 4H), 3.58 – 3.44 (m, 8H), 2.45 (dt, J = 9.2, 6.8 Hz, 4H), 2.33 (t, J = 7.0 Hz, 2H), 2.03 – 1.93 (m, 2H), 1.84 – 1.72 (m, 2H), 1.64 (dt, J = 14.2, 6.9 Hz, 2H), 1.51 – 1.42 (m, 2H), 1.42 – 1.33 (m, 2H).
HRMS (ESI): calculated for $[M+H]^+$, 338.1729; found, 338.1723.

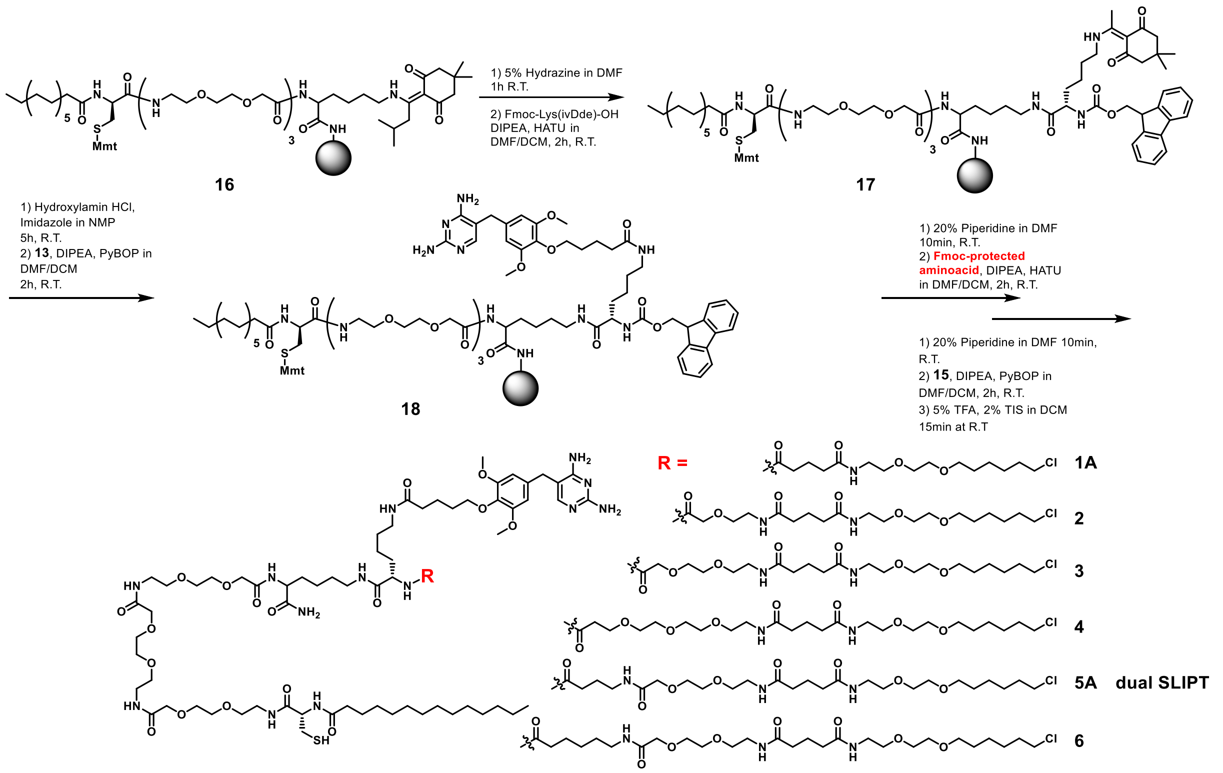

Synthesis of (18)

Synthetic procedure for compound **16** was adapted from (Yoshii et al. 2021).

Compound 16 (0,75 µmol/mg, 120g, 0.1mmol, 1eq.) was synthesized using a peptide synthesizer (Liberty Blue, CEM) on Sieber Amide Resin (Novabiochem) using standard fmoc-based solid phase peptide synthesis. To this end, fmoc-Lys(ivDde)-OH, fmoc-8-amino-3,6-dioxaoctanoid-acid, fmoc-D-Cys(Mmt)-OH, as well as myristic acid were used as building blocks. Fmoc deprotection was performed using 20% piperidine in DMF for 10 min at rt. Coupling was performed automatedly using a mixture of N-terminally fmoc-protected amino acid (0.2M), Oxyma (1M) and DIC (1M) in peptide synthesis grade DMF (Acros Organics). Reaction monitoring was performed by cleaving a small amount of peptide off the resin, using a freshly prepared solution of 5% (v/v) TFA (50µL), and 2% (v/v) TIS (20µL) in DCM (930ul), and performing an analytical LC-MS.

The resin was pre-swelled, according to the producer manual, in 1/1 DMF/DCM for 15 min at rt, before initial deprotection, and successive coupling was performed on the synthesizer. The crude **compound 16** was obtained, attached to the Resin, without notable side product formation, as determined by analytical LC-MS. It was thereafter transferred into a syringe outfitted with filter and a syringe pressure cap for the purpose of manual modification.

Thereafter, all steps were conducted manually. Manual couplings were performed after pre-swelling the resin in DCM. Simultaneously, 2 eq. of the compound to be coupled was stirred in a 50% (v/v) DCM in DMF solution (1ml/0.02 mmol peptide) with 4 eq. of DIPEA, and 1.8 eq. of coupling reagent (HATU, or PyBOP, as specified). Manual fmoc deprotection was performed using 20% piperidine in DMF for 10 min at rt. After every reaction step the resin was thoroughly rinsed with DMF first, then DCM. Final cleavage, and simultaneous Mmt-deprotection of *D*-Cysteine was conducted, using a fresh cleavage cocktail of 5% (v/v) TFA (50µL), and 2% (v/v) TIS (20µL) in DCM (930ul) for 15 min at rt, which was repeated as necessary. Purification of **compound**s **1-8** was performed on a reverse-phase HPLC C4 column (HiChrom).

ivDde-deprotection of **compound 16** (0.1mmol, 1 eq.) was performed using Hydrazine monohydrate in DMF (final concentration 5% (v/v)) for 1h at rt under gentle agitation. After washing, fmoc-lys(Dde)-OH (106.4mg, 0.2mmol, 2 eq.), was chosen for its ability to be partially Dde-deprotected, while retaining Nα-fmoc-protection. The amino acid was dissolved in a 50% (v/v) DCM in DMF solution (3 mL), and coupled using HATU (68.5mg, 0.18mmol, 1.8 eq.) were added. The reaction mixture was stirred at rt for 5 minutes, before adding to the swelled resin. The reaction mixture was gently agitated for 2h and an analytical LC-MS was performed to verify quantitative coupling to afford **compound 17**.

A Dde-deprotection cocktail was prepared, containing hydroxylamine hydrochloride (10.4 mg, 150 µmol, 1.5 eq.), and Imidazole (8.26 µL, 125 µmol, 1.25eq.) in 1-methyl-2-pyrrolidinone (1ml / 0.1mmol resin). Immediately prior to the deprotection, the cocktail was diluted 5/1 with DCM (1ml Dde-deprotection cocktail, 0,2ml DCM) for better efficiency of polysterol-type resin deprotection, and added to the resin. The Resin-attached **compound 17** (0.1mmol, 1 eq.) was deprotected for 5h, or until approximately 60% of **compound 17** was deprotected. The efficacy was determined by LC-MS monitoring. The deprotected compound was washed thoroughly, before drying under reduced pressure for the purpose of aliquoting and derivatisation.

To a stirred solution of **13** (15.1 mg, 40 µmol, 2 eq.) in 50% (v/v) DCM in DMF solution (1 mL), DIPEA and PYBOP were added. After 5 minutes, the mixture was added to the Dde-deprotected peptide (24mg, 0.02mmol, 1eq.) and gently agitated for 2h to afford **compound 18**, coupled to the Resin. LC-MS indicated complete conversion of the Dde-deprotected starting material, and the crude **compound 18** was used without purification in the following steps.

Synthesis of m^{D}cTMP-HTL^{5} (1A)

Pre-swelled **compound 18** (0,75 µmol/mg, 0.03mmol, 1 eq.) was fmoc-deprotected, before thorough washing. **15** (13.4 mg, 60 µmol, 2 eq.) was dissolved in 50% (v/v) DCM in DMF, to which DIPEA and PYBOP were added. The reaction mixture was stirred for 5 minutes at rt before adding to the resin. Thereafter, the reaction was gently agitated at rt for 2h, before draining, and thorough washing. The crude product was cleaved off the resin, dried, and purified, using a linear gradient of 45-65% ACN in H_2O (0.1% TFA). After evaporation of the solvent, the purified product **(1A)** was obtained (4.2mg, 8.23%).

HRMS (ESI): calculated for $[M+H]^+$, 1699.9721; found, 1699.9701; calculated for $[M+2H]^{2+}$, 850.4897; found, 850.4890.

Synthesis of m^{D}cTMP-HTL^{11} (2)

Pre-swelled **compound 18** (0,75 µmol/mg, 0.02 mmol, 1 eq.) was fmoc-deprotected, before thorough washing. 2-(2-((((9*H*-fluoren-9-yl)methoxy)carbonyl)amino)ethoxy)acetic acid (13.7 mg, 40 µmol, 2 eq.) was weighed in in a separate vial, dissolved in 50% (v/v) DCM in, to which DIPEA and HATU were added. The reaction mixture was stirred for 5 minutes at rt before adding to the resin. Thereafter, the reaction was gently agitated at rt for 2h, before draining, and thorough washing.

The peptide (0,75 µmol/mg, 0.02 mmol, 1 eq.) was again fmoc-deprotected and rinsed, before **15** (13.5 mg, 40 µmol, 2 eq.) was dissolved in 50% (v/v) DCM in DMF, to which DIPEA and PYBOP were added. The reaction mixture was stirred for 5 minutes at rt before adding to the resin. Thereafter, the reaction was gently agitated at rt for 2h, before draining, and thorough washing. The crude product was cleaved off the resin, dried, and purified, using a linear gradient of 30-70% ACN in H_2O (0.1% TFA). After evaporation of the solvent, the purified product **(2)** was obtained (0.5mg, 1.39%).

HRMS (ESI): calculated for $[M+3H]^{3+}$, 601.0115; found, 601.0122.

Synthesis of m^{D}cTMP-HTL^{14} (3)

Pre-swelled **compound 18** (0,75 µmol/mg, 0.02 mmol, 1 eq.) was fmoc-deprotected, before thorough washing. Fmoc-8-amino-3,6-dioxaoctanoic acid (15.4 mg, 40 µmol, 2 eq.) was weighed in in a separate vial, dissolved in 50% (v/v) DCM in DMF, to which DIPEA and HATU were added. The reaction mixture was stirred for 5 minutes at rt before adding to the resin. Thereafter, the reaction was gently agitated at rt for 2h, before draining, and thorough washing.

The peptide (0,75 µmol/mg, 0.02 mmol, 1 eq.) was again fmoc-deprotected and rinsed, before **15** (13.5 mg, 40 µmol, 2 eq.) was dissolved in 50% (v/v) DCM in DMF, to which DIPEA and PYBOP were added. The reaction mixture was stirred for 5 minutes at rt before adding to the resin. Thereafter, the reaction was gently agitated at rt for 2h, before draining, and thorough washing. The crude product was cleaved off the resin, dried, and purified, using a linear gradient of 40-70% ACN in H_2O (0.1% TFA). After evaporation of the solvent, the purified product **(3)** was obtained (1.5mg, 3.94%).

HRMS (ESI): calculated for $[M+2H]^{2+}$, 923.0267; found, 923.0257, $[M+3H]^{3+}$, 616.0212; found, 616.0205.

Synthesis of m^{D}cTMP-HTL^{18} (4)

Pre-swelled **compound 18** (0,75 µmol/mg, 0.02 mmol, 1 eq.) was fmoc-deprotected, before thorough washing. 1-(9*H*-fluoren-9-yl)-3-oxo-2,7,10,13-tetraoxa-4-azahexadecan-16-oic acid (17.2 mg, 40 µmol, 2 eq.) was weighed in in a separate vial, dissolved in 50% (v/v) DCM in DMF, to which DIPEA and HATU were added. The reaction mixture was stirred for 5 minutes at rt before adding to the resin. Thereafter, the reaction was gently agitated at rt for 2h, before draining, and thorough washing.

The peptide (0,75 µmol/mg, 0.02 mmol, 1 eq.) was again fmoc-deprotected and rinsed, before **15** (13.5 mg, 40 µmol, 2 eq.) was dissolved in 50% (v/v) DCM in DMF, to which DIPEA and PYBOP were added. The reaction mixture was stirred for 5 minutes at rt before adding to the resin. Thereafter, the reaction was gently agitated at rt for 2h, before draining, and thorough washing the crude product was cleaved off the resin, dried, and purified, using a linear gradient of 30-70% ACN in H_2O (0.1% TFA). After evaporation of the solvent, the purified product **(4)** was obtained (0.4mg, 1.16%).

HRMS (ESI): calculated for $[M+3H]^{3+}$, 635.0341; found, 635.0349.

Synthesis of m^{D}cTMP-HTL^{19}, dual SLIPT (5A)

Pre-swelled **compound 18** (0,75 µmol/mg, 0.02 mmol, 1 eq.) was fmoc-deprotected, before thorough washing. Fmoc-4-aminobutyric acid (13 mg, 40 µmol, 2 eq.) was weighed in in a separate vial, dissolved in 50% (v/v) DCM in DMF, to which DIPEA and HATU were added. The reaction mixture was stirred for 5 minutes at rt before adding to the resin. Thereafter, the reaction was gently agitated at rt for 2h, before draining, and thorough washing. Then, fmoc-8-amino-3,6-dioxaoctanoic acid (15.4 mg, 40 µmol, 2 eq.) was weighed in, dissolved in 50% (v/v) DCM in DMF, to which DIPEA and HATU were added.

The reaction mixture was stirred for 5 minutes at rt before adding to the resin. The reaction was gently agitated at rt for 2h, before draining, and thorough washing.

The peptide (0,75 µmol/mg, 0.02 mmol, 1 eq.) was again fmoc-deprotected and rinsed, before **15** (13.5 mg, 40 µmol, 2 eq.) was dissolved in 50% (v/v) DCM in DMF, to which DIPEA and PYBOP were added. The reaction mixture was stirred for 5 minutes at rt before adding to the resin. Thereafter, the reaction was gently agitated at rt for 2h, before draining, and thorough washing. The crude product was cleaved off the resin, dried, and purified, using a linear gradient of 45-70% ACN in H_2O (0.1% TFA). After evaporation of the solvent, the purified product **(5A)** was obtained (0.1mg, 0.68%).

HRMS (ESI): calculated for $[M+3H]^{3+}$, 644.0378; found, 644.0385.

Synthesis of m^{D}cTMP-HTL[21] (6)

Pre-swelled **compound 18** (0,75 µmol/mg, 0.02 mmol, 1 eq.) was fmoc-deprotected, before thorough washing. Fmoc-6-aminocaproic acid (14.1 mg, 40 µmol, 2 eq.), was weighed in in a separate vial, dissolved in 50% (v/v) DCM in DMF, to which DIPEA and HATU were added. The reaction mixture was stirred for 5 minutes at rt before adding to the resin. Thereafter, the reaction was gently agitated at rt for 2h, before draining, and thorough washing.

Then, fmoc-8-amino-3,6-dioxaoctanoic acid (15.4 mg, 40 µmol, 2 eq.) was weighed in, dissolved in 50% (v/v) DCM in DMF, to which DIPEA and HATU were added. The reaction mixture was stirred for 5 minutes at rt before adding to the resin. The reaction was gently agitated at rt for 2h, before draining, and thorough washing.

The peptide (0,75 µmol/mg, 0.02 mmol, 1 eq.) was again fmoc-deprotected and rinsed, before **15** (13.5 mg, 40 µmol, 2 eq.) was dissolved in 50% (v/v) DCM in DMF, to which DIPEA and PYBOP were added. The reaction mixture was stirred for 5 minutes at rt before adding to the resin. Thereafter, the reaction was gently agitated at rt for 2h, before draining, and thorough washing. The crude product was cleaved off the resin, dried, and purified, using a linear gradient of 42-48% ACN in H_2O (0.1% TFA). After evaporation of the solvent, the purified product **(6)** was obtained (0.1mg, 0.32%).

HRMS (ESI): calculated for $[M+3H]^{3+}$, 653.3815; found, 653.3826.

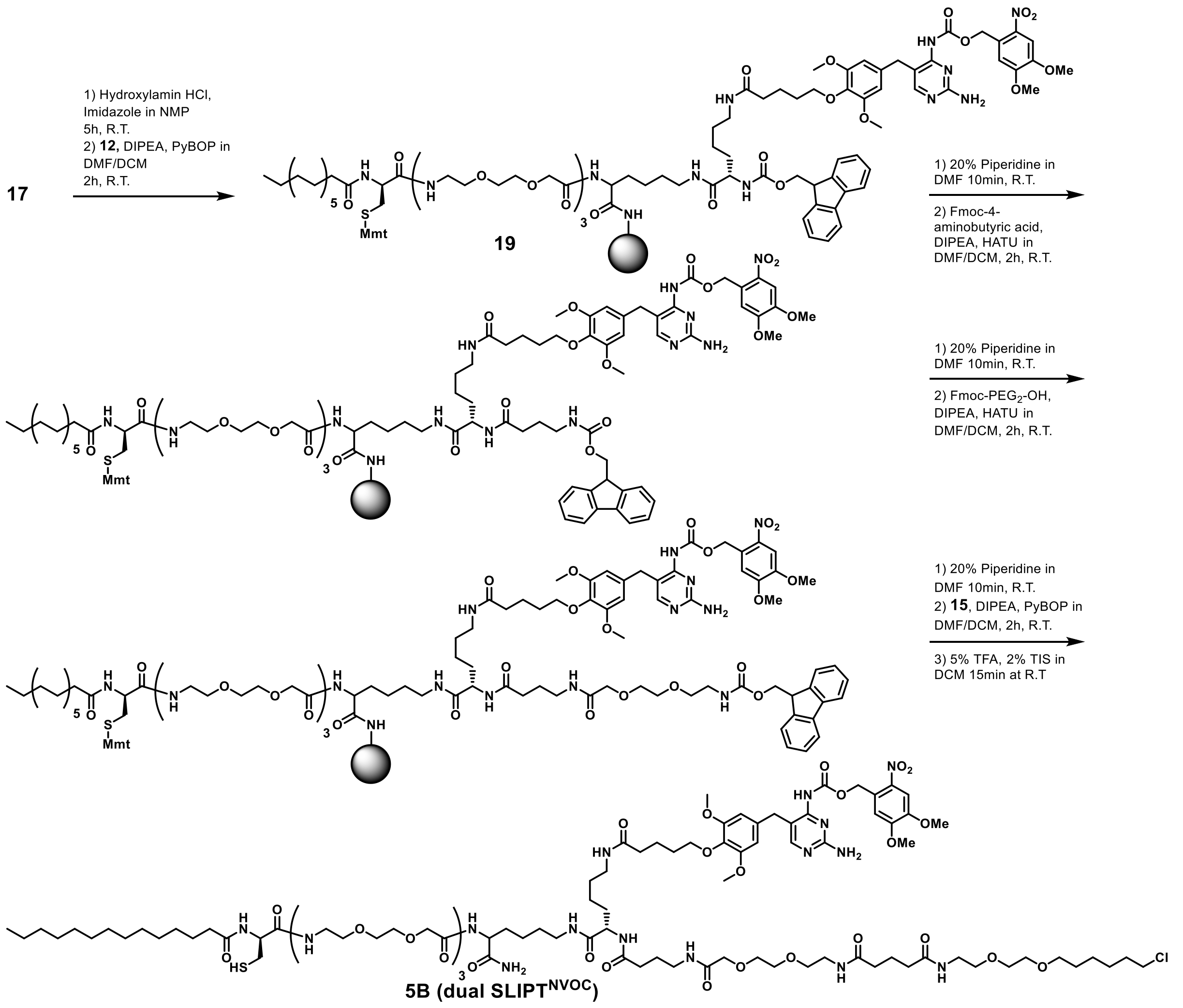

Synthesis of m^{D}cTMP^{NVOC}-HTL^{19}, dual SLIPT^{NVOC} (5B)

Synthesis of **17** and subsequent Dde-deprotection were conducted as described for **(17)**. To a stirred solution of **12** (24.6 mg, 40 µmol, 2 eq.) in 50% (v/v) DCM in DMF solution (1 mL), DIPEA (13.2 µL, 80 µmol, 4 eq.) and PYBOP (18.7 mg, 36 µmol, 1.8 eq.) were added. After 5 minutes, the mixture was added to the Dde-deprotected peptide **17** (24mg, 0.02mmol, 1eq.) and gently agitated for 2h.

The TMP^{NVOC}-coupled **compound 19** (0,75 µmol/mg, 0.02 mmol, 1 eq.) was fmoc-deprotected, before thorough washing. Fmoc-4-aminobutyric acid (13 mg, 40 µmol, 2 eq.) was weighed in in a separate vial, dissolved in 50% (v/v) DCM in DMF (1 mL), to which DIPEA (13.2 µL, 80 µmol, 4 eq.) and HATU (13.7 mg, 36 µmol, 1.8 eq.) were added. The reaction mixture was stirred for 5 minutes at rt before adding to the resin. Thereafter, the reaction was gently agitated at rt for 2h, before draining, and thorough washing. Then, fmoc-8-amino-3,6-dioxaoctanoic acid (15.4 mg, 40 µmol, 2 eq.) was weighed in, dissolved in 50% (v/v) DCM in DMF (1 mL), to which DIPEA (13.2 µL, 80 µmol, 4 eq.) and HATU (13.7 mg, 36 µmol, 1.8 eq.) were added. The reaction mixture was stirred for 5 minutes at rt before adding to the resin. The reaction was gently agitated at rt for 2h, before draining, and thorough washing.

The peptide (0,75 µmol/mg, 0.02 mmol, 1 eq.) was again fmoc-deprotected and rinsed, before **15** (13.5 mg, 40 µmol, 2 eq.) was dissolved in 50% (v/v) DCM in DMF (1mL), to which DIPEA (13.2 µL, 80 µmol, 4 eq.) and PYBOP (18.7 mg, 36 µmol, 1.8 eq.) were added. The reaction mixture was stirred for 5 minutes at rt before adding to the resin. Thereafter, the reaction was gently agitated at rt for 2h, before draining, and thorough washing. The crude product was cleaved off the resin, dried, and purified, using

a linear gradient of 55-65% ACN (0.1% FA) in H_2O (0.1% FA). After evaporation of the solvent, the purified product **(5B)** was obtained (0.1mg, 0.68%).

HRMS (ESI): calculated for $[M+3H]^{3+}$, 723.7188; found, 723.7187.

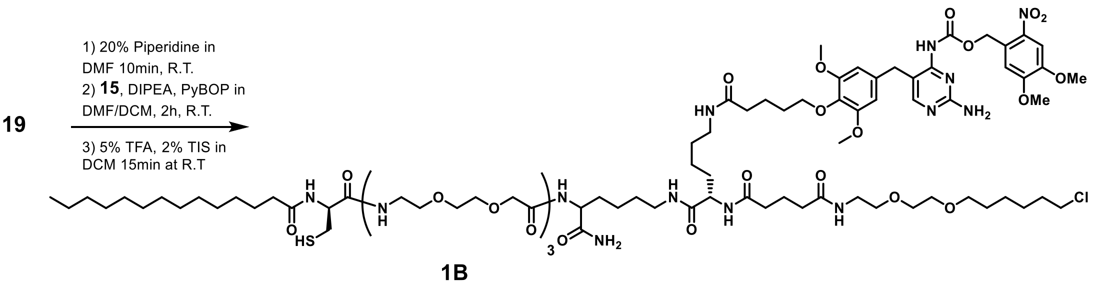

Synthesis of $m^{D}cTMP^{NVOC}$-HTL^{5} (1B)

15 (6.76 mg, 20 µmol, 2 eq.) was weighed in and dissolved in 50% (v/v) DCM in DMF (0.5 mL). DIPEA (6.61 µL, 40 µmol, 4 eq.) and PYBOP (9.37 mg, 18 µmol, 1.8 eq.) were added successively and the reaction mixture was stirred for 5 min at rt, before adding into the deprotected, pre-swelled **compound 19** (0,75 µmol/mg, 10 µmol, 1 eq.). The reaction was gently agitated for 2h under exclusion of light, before draining, thorough washing, and cleaving the crude off the resin as described for compounds **1-6**. The solvent was evaporated and the crude product purified using a linear gradient of 30-70% ACN in H_2O (0.1% TFA), yielding the purified product (0.07mg, 3.48%).

HRMS (ESI): calculated for $[M+2H]^{2+}$, 977.0190; found, 977.0183.

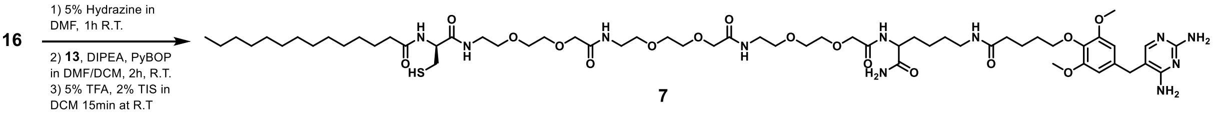

Synthesis of $m^{D}cTMP$ (7)

Synthetic procedure for **compound 16** was adapted from (Nakamura et al. 2020b), and deprotection conducted as described for (**16**).

To a stirring solution of 13 (15.1 mg, 40 µmol, 2 eq.) in 50% (v/v) DCM in DMF (1 mL), DIPEA (13.2 µL, 80 µmol, 4 eq.) and PYBOP (18.7 mg, 36 µmol, 1.8 eq.) were added at rt. After 5 minutes, the mixture was added to pre-swelled **compound 16** (12.5mg, 0.02mmol, 1eq.) and gently agitated for 2h. The resin was thoroughly washed, and dried. Thereafter, the crude was cleaved off the resin, as described above for compounds (**1-6**). The solvent was evaporated and the crude product purified using a linear gradient of 5-80% ACN in H_2O (0.1% TFA), yielding the purified product (1.45mg, 6%). The spectroscopic data are in agreement with the literature.

HRMS (ESI): calculated for $[M+H]^{1+}$, 1252.7222; found, 1252.7218; calculated for $[M+2H]^{2+}$, 626.8647; found, 626.8642.

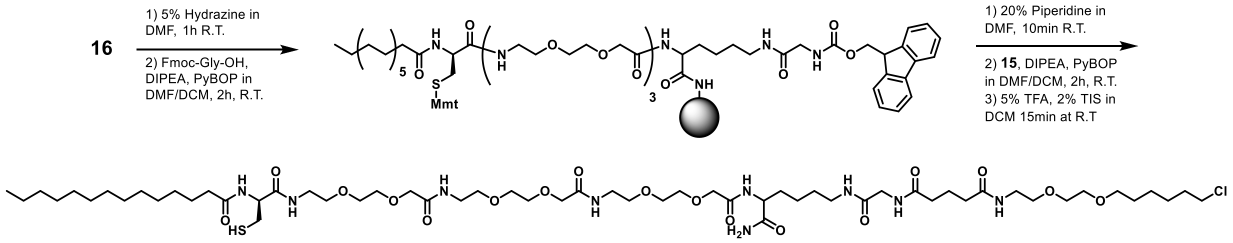

8

Synthesis of m^{D}cgHTL (8)

Compound 8 represents an optimization of the previously reported mgcHTL (Nakamura et al. 2020a).

Compound 16 was prepared, and deprotected as described for **(16)**. Fmoc-glycine (23.8 mg, 80 µmol, 2 eq.) was weighed in and dissolved in 50% (v/v) DCM in DMF (1mL). DIPEA (26.4 µL, 160 µmol, 4 eq.) and PYBOP (37.5 mg, 72 µmol, 1.8 eq.) were added successively and the reaction mixture was stirred for 5 min at rt, before adding into the deprotected, pre-swelled **compound 16** (0,75 µmol/mg, 40 µmol, 1 eq.). The reaction was gently agitated for 2h, before draining, and washing. The crude was used for further steps without purification.

The peptide (0,75 µmol/mg, 0.04 mmol, 1 eq.) was again fmoc-deprotected and rinsed, before **15** (27 mg, 80 µmol, 2 eq.) was dissolved in 50% (v/v) DCM in DMF (2mL), to which DIPEA (26.4 µL, 160 µmol, 4 eq.) and PYBOP (37.5 mg, 72 µmol, 1.8 eq.) were added. The reaction mixture was stirred for 5 minutes at rt before adding to the resin. Thereafter, the reaction was gently agitated at rt for 2h, before draining, thorough washing, and cleaving the crude off the resin as described for compounds **1-6**. The solvent was evaporated and the crude product purified using a linear gradient of 50-70% ACN (0.1% FA) in H_2O (0.1% FA), yielding the purified product (1.45mg, 2.85%).

HRMS (ESI): calculated for $[M+2H]^{2+}$, 635.8709; found, 635.8702.

Supplementary Movie

Movie S1 Sequential dimerization of ^{iK6}eDHFR-mEGFP and HOB-mScar, induced by incubation with 10 μM **dual SLIPT$^{\mathbf{NVOC}}$** and subsequent irradiation with single cell resolution. (time-lapse movie of **Figure 4, S3**) Scale bar, 10 μm.

cDNA

Plasmid construction was conducted by PCR-amplification of the coding sequences, as well as the plasmid backbone, elongated with primers purchased from Sigma Aldrich. The PCR product was DpnI-digested (Thermo Scientific), and purified using the QIAquick PCR Purification Kit (Qiagen) before Gibson Assembly was performed, using a 1to 1 ratio of backbone and insert. Sequence validation was performed by Eurofins (Sanger Sequencing). Point mutations were introduced using the Q5® Site-Directed Mutagenesis Kit (New England Biolabs).

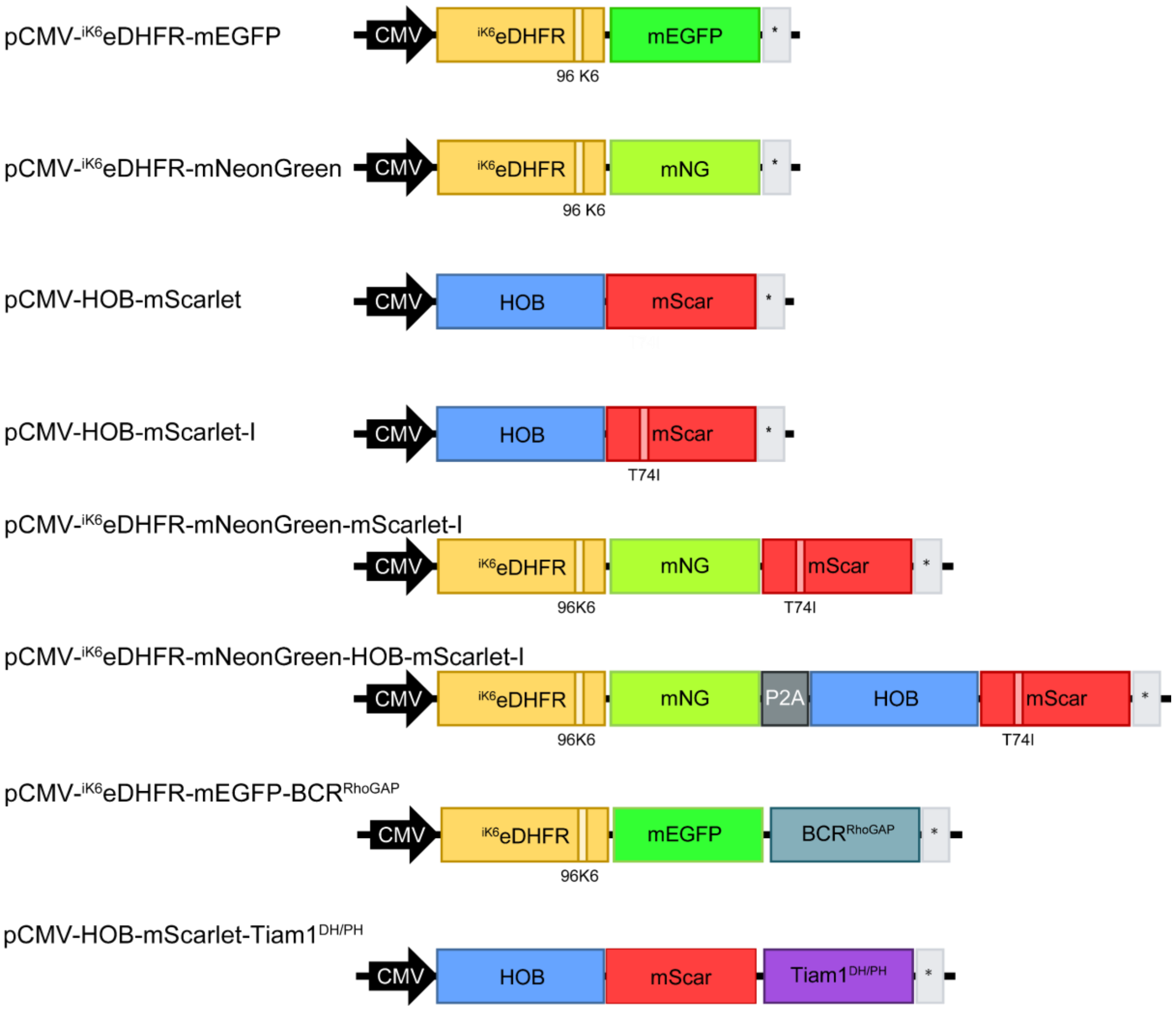

Supplementary Sequences

pCMV-^{iK6}eDHFR-mEGFP

> Amino acid sequence

MISLIAALAVDRVIGMENAMPWNLPADLAWFKRNTLNKPVIMGRHTWESIGRPLPGRKNIILSSQPGTDKKKKKK
DRVTWVKSVDEAIAACGDVPEIMVIGGGRVYEQFLPKAQKLYLTHIDAEVEGDTHFPDYEPDDWESVFSEFHDAD
AQNSHSYCFEILERRSGSGDPPVATMMVSKGEELFTGVVPILVELDGDVNGHKFSVSGEGEGDATYGKLTLKFIC
TTGKLPVPWPTLVTTLTYGVQCFSRYPDHMKQHDFFKSAMPEGYVQERTIFFKDDGNYKTRAEVKFEGDTLVNRI
ELKGIDFKEDGNILGHKLEYNYNSHNVYIMADKQKNGIKVNFKIRHNIEDGSVQLADHYQQNTPIGDGPVLLPDN
HYLSTQSKLSKDPNEKRDHMVLLEFVTAAGITLGMDELYK

> DNA Sequence

ATGATCAGTCTGATTGCGGCGTTAGCGGTAGATCGCGTTATCGGCATGGAAAACGCCATGCCGTGGAACCTGCCT
GCCGATCTCGCCTGGTTTAAACGCAACACCTTAAATAAACCCGTGATTATGGGCCGCCATACCTGGGAATCAATC
GGTCGTCCGTTGCCAGGACGCAAAAATATTATCCTCAGCAGTCAACCGGGTACGGACAAAAAAAAGAAAAAGAAA
GATCGCGTAACGTGGGTGAAGTCGGTGGATGAAGCCATCGCGGCGTGTGGTGACGTACCAGAAATCATGGTGATT
GGCGGCGGTCGCGTTTATGAACAGTTCTTGCCAAAAGCGCAAAAACTGTATCTGACGCATATCGACGCAGAAGTG
GAAGGCGACACCCATTTCCCGGATTACGAGCCGGATGACTGGGAATCGGTATTCAGCGAATTCCACGATGCTGAT
GCGCAGAACTCTCACAGCTATTGCTTTGAGATTCTGGAGCGGCGGAGCGGCTCCGGGGATCCACCGGTCGCCACC
ATGATGGTGAGCAAGGGCGAGGAGCTGTTCACCGGGGTGGTGCCCATCCTGGTCGAGCTGGACGGCGACGTAAAC
GGCCACAAGTTCAGCGTGTCCGGCGAGGGCGAGGGCGATGCCACCTACGGCAAGCTGACCCTGAAGTTCATCTGC
ACCACCGGCAAGCTGCCCGTGCCCTGGCCCACCCTCGTGACCACCCTGACCTACGGCGTGCAGTGCTTCAGCCGC
TACCCCGACCACATGAAGCAGCACGACTTCTTCAAGTCCGCCATGCCCGAAGGCTACGTCCAGGAGCGCACCATC
TTCTTCAAGGACGACGGCAACTACAAGACCCGCGCCGAGGTGAAGTTCGAGGGCGACACCCTGGTGAACCGCATC
GAGCTGAAGGGCATCGACTTCAAGGAGGACGGCAACATCCTGGGGCACAAGCTGGAGTACAACTACAACAGCCAC
AACGTCTATATCATGGCCGACAAGCAGAAGAACGGCATCAAGGTGAACTTCAAGATCCGCCACAACATCGAGGAC
GGCAGCGTGCAGCTCGCCGACCACTACCAGCAGAACACCCCCATCGGCGACGGCCCCGTGCTGCTGCCCGACAAC
CACTACCTGAGCACCCAGTCCAAACTGAGCAAAGACCCCAACGAGAAGCGCGATCACATGGTCCTGCTGGAGTTC
GTGACCGCCGCCGGGATCACTCTCGGCATGGACGAGCTGTACAAGTAG

^{iK6}eDHFR 6x internal Lysine Linker mEGFP

pCMV-^{iK6}eDHFR-mNeonGreen

> Amino acid sequence

MISLIAALAVDRVIGMENAMPWNLPADLAWFKRNTLNKPVIMGRHTWESIGRPLPGRKNIILSSQPGTDKKKKKK
DRVTWVKSVDEAIAACGDVPEIMVIGGGRVYEQFLPKAQKLYLTHIDAEVEGDTHFPDYEPDDWESVFSEFHDAD
AQNSHSYCFEILERRSGSGDPPVATMVSKGEEDNMASLPATHELHIFGSINGVDFDMVGQGTGNPNDGYEELNLK
STKGDLQFSPWILVPHIGYGFHQYLPYPDGMSPFQAAMVDGSGYQVHRTMQFEDGASLTVNYRYTYEGSHIKGEA
QVKGTGFPADGPVMTNSLTAADWCRSKKTYPNDKTIISTFKWSYTTGNGKRYRSTARTTYTFAKPMAANYLKNQP
MYVFRKTELKHSKTELNFKEWQKAFTDVMGMDELY

> DNA Sequence

ATGATCAGTCTGATTGCGGCGTTAGCGGTAGATCGCGTTATCGGCATGGAAAACGCCATGCCGTGGAACCTGCCT
GCCGATCTCGCCTGGTTTAAACGCAACACCTTAAATAAACCCGTGATTATGGGCCGCCATACCTGGGAATCAATC
GGTCGTCCGTTGCCAGGACGCAAAAATATTATCCTCAGCAGTCAACCGGGTACGGACAAAAAAAAGAAAAAGAAA
GATCGCGTAACGTGGGTGAAGTCGGTGGATGAAGCCATCGCGGCGTGTGGTGACGTACCAGAAATCATGGTGATT
GGCGGCGGTCGCGTTTATGAACAGTTCTTGCCAAAAGCGCAAAAACTGTATCTGACGCATATCGACGCAGAAGTG
GAAGGCGACACCCATTTCCCGGATTACGAGCCGGATGACTGGGAATCGGTATTCAGCGAATTCCACGATGCTGAT
GCGCAGAACTCTCACAGCTATTGCTTTGAGATTCTGGAGCGGCGGAGCGGCTCCGGGGATCCACCGGTCGCCACC
ATGGTGAGCAAGGGCGAGGAGGATAACATGGCCTCTCTCCCAGCGACACATGAGTTACACATCTTTGGCTCCATC
AACGGTGTGGACTTTGACATGGTGGGTCAGGGCACCGGCAATCCAAATGATGGTTATGAGGAGTTAAACCTGAAG
TCCACCAAGGGTGACCTCCAGTTCTCCCCCTGGATTCTGGTCCCTCATATCGGGTATGGCTTCCATCAGTACCTG
CCCTACCCTGACGGGATGTCGCCTTTCCAGGCCGCCATGGTAGATGGCTCCGGATACCAAGTCCATCGCACAATG
CAGTTTGAAGATGGTGCCTCCCTTACTGTTAACTACCGCTACACCTACGAGGGAAGCCACATCAAAGGAGAGGCC
CAGGTGAAGGGGACTGGTTTCCCTGCTGACGGTCCTGTGATGACCAACTCGCTGACCGCTGCGGACTGGTGCAGG
TCGAAGAAGACTTACCCCAACGACAAAACCATCATCAGTACCTTTAAGTGGAGTTACACCACTGGAAATGGCAAG
CGCTACCGGAGCACTGCGCGGACCACCTACACCTTTGCCAAGCCAATGGCGGCTAACTATCTGAAGAACCAGCCG
ATGTACGTGTTCCGTAAGACGGAGCTCAAGCACTCCAAGACCGAGCTCAACTTCAAGGAGTGGCAAAAGGCCTTT
ACCGATGTGATGGGCATGGACGAGCTGTAC

^{iK6}eDHFR 6x internal Lysine Linker mNeonGreen

pCMV-HOB-mScarlet

> Amino acid sequence

MIGTGFPFDPHYVEVLGERMHYVDVGPRDGTPVLFLHGNPTSSYVWRNIIPHVAPTHRCIAPDLIGMGKSDKPDL
GYFFDDHVRFMDAFIEALGLEEVVLVIHDWGSALGFHWAKRNPERVKGIAFMEFIRPIPTWDEWPKFARKTFQAF
RTKKVGRKLIIDQNVFIEGTLPMGVVRPLTEVEMDHYREPFLNPVDREPLWRFPNELPIAGEPANIVALVEEYMD
WLHQSPVPKLLFWGTPGVLIPPAEAARLAKSLPNCKAVDIGPGLNLLQEDNPDLIGSEIARWLSTLEISGMVSKG
EAVIKEFMRFKVHMEGSMNGHEFEIEGEGEGRPYEGTQTAKLKVTKGGPLPFSWDILSPQFMYGSRAFTKHPADI
PDYYKQSFPEGFKWERVMNFEDGGAVTVTQDTSLEDGTLIYKVKLRGTNFPPDGPVMQKKTMGWEASTERLYPED
GVLKGDIKMALRLKDGGRYLADFKTTYKAKKPVQMPGAYNVDRKLDITSHNEDYTVVEQYERSEGRHSTGGMDEL
YK

> DNA Sequence

ATGATTGGCACCGGTTTTCCGTTTGATCCGCATTATGTTGAAGTTCTGGGTGAACGTATGCATTATGTGGATGTT
GGTCCGCGTGATGGTACACCGGTTCTGTTTCTGCATGGTAATCCGACCAGCAGCTATGTTTGGCGTAACATTATT
CCGCATGTTGCACCGACCCATCGTTGTATTGCACCGGATCTGATTGGTATGGGTAAAAGCGATAAACCTGATCTG
GGCTATTTTTTCGATGATCATGTGCGTTTTATGGACGCCTTTATTGAAGCACTGGGTCTGGAAGAAGTTGTGCTG
GTTATTCATGATTGGGGTAGCGCACTGGGTTTTCATTGGGCAAAACGTAATCCGGAACGTGTTAAAGGTATTGCC
TTTATGGAATTTATTCGTCCGATTCCGACCTGGGATGAATGGCCGAAATTTGCACGTAAAACCTTTCAGGCATTT
CGCACCAAAAAAGTTGGTCGCAAACTGATTATTGACCAGAACGTTTTTATCGAAGGCACCCTGCCGATGGGTGTT
GTTCGTCCGCTGACCGAAGTTGAAATGGATCATTATCGTGAACCGTTTCTGAATCCGGTTGATCGCGAACCGCTG
TGGCGTTTTCCGAATGAACTGCCGATTGCCGGTGAACCTGCAAATATTGTTGCACTGGTTGAAGAGTATATGGAT
TGGCTGCATCAGAGTCCGGTTCCGAAACTGCTGTTTTGGGGCACACCGGGTGTTCTGATTCCGCCTGCAGAAGCA
GCACGTCTGGCAAAAAGCCTGCCGAATTGTAAAGCAGTTGATATTGGTCCGGGTCTGAATCTGCTGCAAGAAGAT
AATCCAGATCTGATCGGTAGTGAAATTGCACGTTGGCTGAGCACCCTGGAAATTAGCGGCATGGTGAGCAAGGGC
GAGGCAGTGATCAAGGAGTTCATGCGGTTCAAGGTGCACATGGAGGGCTCCATGAACGGCCACGAGTTCGAGATC
GAGGGCGAGGGCGAGGGCCGCCCCTACGAGGGCACCCAGACCGCCAAGCTGAAGGTGACCAAGGGTGGCCCCCTG
CCCTTCTCCTGGGACATCCTGTCCCCTCAGTTCATGTACGGCTCCAGGGCCTTCACCAAGCACCCCGCCGACATC
CCCGACTACTATAAGCAGTCCTTCCCCGAGGGCTTCAAGTGGGAGCGCGTGATGAACTTCGAGGACGGCGGCGCC
GTGACCGTGACCCAGGACACCTCCCTGGAGGACGGCACCCTGATCTACAAGGTGAAGCTCCGCGGCACCAACTTC
CCTCCTGACGGCCCCGTAATGCAGAAGAAGACAATGGGCTGGGAAGCGTCCACCGAGCGGTTGTACCCCGAGGAC
GGCGTGCTGAAGGGCGACATTAAGATGGCCCTGCGCCTGAAGGACGGCGGCCGCTACCTGGCGGACTTCAAGACC
ACCTACAAGGCCAAGAAGCCCGTGCAGATGCCCGGCGCCTACAACGTCGACCGCAAGTTGGACATCACCTCCCAC
AACGAGGACTACACCGTGGTGGAACAGTACGAACGCTCCGAGGGCCGCCACTCCACCGGCGGCATGGACGAGCTG
TACAAGTAG

HOB mScarlet

pCMV-HOB-mScarlet-I

> Amino acid sequence

MIGTGFPFDPHYVEVLGERMHYVDVGPRDGTPVLFLHGNPTSSYVWRNIIPHVAPTHRCIAPDLIGMGKSDKPDL
GYFFDDHVRFMDAFIEALGLEEVVLVIHDWGSALGFHWAKRNPERVKGIAFMEFIRPIPTWDEWPKFARKTFQAF
RTKKVGRKLIIDQNVFIEGTLPMGVVRPLTEVEMDHYREPFLNPVDREPLWRFPNELPIAGEPANIVALVEEYMD
WLHQSPVPKLLFWGTPGVLIPPAEAARLAKSLPNCKAVDIGPGLNLLQEDNPDLIGSEIARWLSTLEISGMVSKG
EAVIKEFMRFKVHMEGSMNGHEFEIEGEGEGRPYEGTQTAKLKVTKGGPLPFSWDILSPQFMYGSRAFIKHPADI
PDYYKQSFPEGFKWERVMNFEDGGAVTVTQDTSLEDGTLIYKVKLRGTNFPPDGPVMQKKTMGWEASTERLYPED
GVLKGDIKMALRLKDGGRYLADFKTTYKAKKPVQMPGAYNVDRKLDITSHNEDYTVVEQYERSEGRHSTGGMDEL
YK

> DNA Sequence

ATGATTGGCACCGGTTTTCCGTTTGATCCGCATTATGTTGAAGTTCTGGGTGAACGTATGCATTATGTGGATGTT
GGTCCGCGTGATGGTACACCGGTTCTGTTTCTGCATGGTAATCCGACCAGCAGCTATGTTTGGCGTAACATTATT
CCGCATGTTGCACCGACCCATCGTTGTATTGCACCGGATCTGATTGGTATGGGTAAAAGCGATAAACCTGATCTG
GGCTATTTTTTCGATGATCATGTGCGTTTTATGGACGCCTTTATTGAAGCACTGGGTCTGGAAGAAGTTGTGCTG
GTTATTCATGATTGGGGTAGCGCACTGGGTTTTCATTGGGCAAAACGTAATCCGGAACGTGTTAAAGGTATTGCC
TTTATGGAATTTATTCGTCCGATTCCGACCTGGGATGAATGGCCGAAATTTGCACGTAAAACCTTTCAGGCATTT
CGCACCAAAAAAGTTGGTCGCAAACTGATTATTGACCAGAACGTTTTTATCGAAGGCACCCTGCCGATGGGTGTT
GTTCGTCCGCTGACCGAAGTTGAAATGGATCATTATCGTGAACCGTTTCTGAATCCGGTTGATCGCGAACCGCTG
TGGCGTTTTCCGAATGAACTGCCGATTGCCGGTGAACCTGCAAATATTGTTGCACTGGTTGAAGAGTATATGGAT
TGGCTGCATCAGAGTCCGGTTCCGAAACTGCTGTTTTGGGGCACACCGGGTGTTCTGATTCCGCCTGCAGAAGCA
GCACGTCTGGCAAAAAGCCTGCCGAATTGTAAAGCAGTTGATATTGGTCCGGGTCTGAATCTGCTGCAAGAAGAT
AATCCAGATCTGATCGGTAGTGAAATTGCACGTTGGCTGAGCACCCTGGAAATTAGCGGCATGGTGAGCAAGGGC
GAGGCAGTGATCAAGGAGTTCATGCGGTTCAAGGTGCACATGGAGGGCTCCATGAACGGCCACGAGTTCGAGATC
GAGGGCGAGGGCGAGGGCCGCCCCTACGAGGGCACCCAGACCGCCAAGCTGAAGGTGACCAAGGGTGGCCCCCTG
CCCTTCTCCTGGGACATCCTGTCCCCTCAGTTCATGTACGGCTCCAGGGCCTTCAUCAAGCACCCCGCCGACATC
CCCGACTACTATAAGCAGTCCTTCCCCGAGGGCTTCAAGTGGGAGCGCGTGATGAACTTCGAGGACGGCGGCGCC
GTGACCGTGACCCAGGACACCTCCCTGGAGGACGGCACCCTGATCTACAAGGTGAAGCTCCGCGGCACCAACTTC
CCTCCTGACGGCCCCGTAATGCAGAAGAAGACAATGGGCTGGGAAGCGTCCACCGAGCGGTTGTACCCCGAGGAC
GGCGTGCTGAAGGGCGACATTAAGATGGCCCTGCGCCTGAAGGACGGCGGCCGCTACCTGGCGGACTTCAAGACC
ACCTACAAGGCCAAGAAGCCCGTGCAGATGCCCGGCGCCTACAACGTCGACCGCAAGTTGGACATCACCTCCCAC
AACGAGGACTACACCGTGGTGGAACAGTACGAACGCTCCGAGGGCCGCCACTCCACCGGCGGCATGGACGAGCTG
TACAAGTAG

HOB mScarlet T74I mutation

pCMV-^{iK6}eDHFR-mNeonGreen-mScarlet-I

> Amino acid sequence

MISLIAALAVDRVIGMENAMPWNLPADLAWFKRNTLNKPVIMGRHTWESIGRPLPGRKNIILSSQPGTDKKKKKK
DRVTWVKSVDEAIAACGDVPEIMVIGGGRVYEQFLPKAQKLYLTHIDAEVEGDTHFPDYEPDDWESVFSEFHDAD
AQNSHSYCFEILERRSGSGDPPVATMVSKGEEDNMASLPATHELHIFGSINGVDFDMVGQGTGNPNDGYEELNLK
STKGDLQFSPWILVPHIGYGFHQYLPYPDGMSPFQAAMVDGSGYQVHRTMQFEDGASLTVNYRYTYEGSHIKGEA
QVKGTGFPADGPVMTNSLTAADWCRSKKTYPNDKTIISTFKWSYTTGNGKRYRSTARTTYTFAKPMAANYLKNQP
MYVFRKTELKHSKTELNFKEWQKAFTDVMGMDELYSGLSKGEEMVSKGEAVIKEFMRFKVHMEGSMNGHEFEIEG
EGEGRPYEGTQTAKLKVTKGGPLPFSWDILSPQFMYGSRAFIKHPADIPDYYKQSFPEGFKWERVMNFEDGGAVT
VTQDTSLEDGTLIYKVKLRGTNFPPDGPVMQKKTMGWEASTERLYPEDGVLKGDIKMALRLKDGGRYLADFKTTY
KAKKPVQMPGAYNVDRKLDITSHNEDYTVVEQYERSEGRHSTGGMDELYK

> DNA Sequence

ATGATCAGTCTGATTGCGGCGTTAGCGGTAGATCGCGTTATCGGCATGGAAAACGCCATGCCGTGGAACCTGCCT
GCCGATCTCGCCTGGTTTAAACGCAACACCTTAAATAAACCCGTGATTATGGGCCGCCATACCTGGGAATCAATC
GGTCGTCCGTTGCCAGGACGCAAAAATATTATCCTCAGCAGTCAACCGGGTACGGACAAAAAAAAGAAAAAGAAA
GATCGCGTAACGTGGGTGAAGTCGGTGGATGAAGCCATCGCGGCGTGTGGTGACGTACCAGAAATCATGGTGATT
GGCGGCGGTCGCGTTTATGAACAGTTCTTGCCAAAAGCGCAAAAACTGTATCTGACGCATATCGACGCAGAAGTG
GAAGGCGACACCCATTTCCCGGATTACGAGCCGGATGACTGGGAATCGGTATTCAGCGAATTCCACGATGCTGAT
GCGCAGAACTCTCACAGCTATTGCTTTGAGATTCTGGAGCGGCGGAGCGGCTCCGGGGATCCACCGGTCGCCACC
ATGGTGAGCAAGGGCGAGGAGGATAACATGGCCTCTCTCCCAGCGACACATGAGTTACACATCTTTGGCTCCATC
AACGGTGTGGACTTTGACATGGTGGGTCAGGGCACCGGCAATCCAAATGATGGTTATGAGGAGTTAAACCTGAAG
TCCACCAAGGGTGACCTCCAGTTCTCCCCCTGGATTCTGGTCCCTCATATCGGGTATGGCTTCCATCAGTACCTG
CCCTACCCTGACGGGATGTCGCCTTTCCAGGCCGCCATGGTAGATGGCTCCGGATACCAAGTCCATCGCACAATG
CAGTTTGAAGATGGTGCCTCCCTTACTGTTAACTACCGCTACACCTACGAGGGAAGCCACATCAAAGGAGAGGCC
CAGGTGAAGGGGACTGGTTTCCCTGCTGACGGTCCTGTGATGACCAACTCGCTGACCGCTGCGGACTGGTGCAGG
TCGAAGAAGACTTACCCCAACGACAAAACCATCATCAGTACCTTTAAGTGGAGTTACACCACTGGAAATGGCAAG
CGCTACCGGAGCACTGCGCGGACCACCTACACCTTTGCCAAGCCAATGGCGGCTAACTATCTGAAGAACCAGCCG
ATGTACGTGTTCCGTAAGACGGAGCTCAAGCACTCCAAGACCGAGCTCAACTTCAAGGAGTGGCAAAAGGCCTTT
ACCGATGTGATGGGCATGGACGAGCTGTACAGCGGCCUAAGCAAAGGCGAAGAAATGGTGAGCAAGGGCGAGGCA
GTGATCAAGGAGTTCATGCGGTTCAAGGTGCACATGGAGGGCTCCATGAACGGCCACGAGTTCGAGATCGAGGGC
GAGGGCGAGGGCCGCCCCTACGAGGGCACCCAGACCGCCAAGCTGAAGGTGACCAAGGGTGGCCCCCTGCCCTTC
TCCTGGGACATCCTGTCCCCTCAGTTCATGTACGGCTCCAGGGCCTTCAUCAAGCACCCCGCCGACATCCCCGAC
TACTATAAGCAGTCCTTCCCCGAGGGCTTCAAGTGGGAGCGCGTGATGAACTTCGAGGACGGCGGCGCCGTGACC
GTGACCCAGGACACCTCCCTGGAGGACGGCACCCTGATCTACAAGGTGAAGCTCCGCGGCACCAACTTCCCTCCT
GACGGCCCCGTAATGCAGAAGAAGACAATGGGCTGGGAAGCGTCCACCGAGCGGTTGTACCCCGAGGACGGCGTG
CTGAAGGGCGACATTAAGATGGCCCTGCGCCTGAAGGACGGCGGCCGCTACCTGGCGGACTTCAAGACCACCTAC
AAGGCCAAGAAGCCCGTGCAGATGCCCGGCGCCTACAACGTCGACCGCAAGTTGGACATCACCTCCCACAACGAG
GACTACACCGTGGTGGAACAGTACGAACGCTCCGAGGGCCGCCACTCCACCGGCGGCATGGACGAGCTGTACAAG
TAG

^{iK6}eDHFR 6x internal Lysine Linker mNeonGreen Linker mScarlet T74I mutation

pCMV-[iK6]eDHFR-mNeonGreen-HOB-mScarlet-I

> Amino acid sequence

MISLIAALAVDRVIGMENAMPWNLPADLAWFKRNTLNKPVIMGRHTWESIGRPLPGRKNIILSSQPGTDKKKKKK
DRVTWVKSVDEAIAACGDVPEIMVIGGGRVYEQFLPKAQKLYLTHIDAEVEGDTHFPDYEPDDWESVFSEFHDAD
AQNSHSYCFEILERRSGSGDPPVATMVSKGEEDNMASLPATHELHIFGSINGVDFDMVGQGTGNPNDGYEELNLK
STKGDLQFSPWILVPHIGYGFHQYLPYPDGMSPFQAAMVDGSGYQVHRTMQFEDGASLTVNYRYTYEGSHIKGEA
QVKGTGFPADGPVMTNSLTAADWCRSKKTYPNDKTIISTFKWSYTTGNGKRYRSTARTTYTFAKPMAANYLKNQP
MYVFRKTELKHSKTELNFKEWQKAFTDVMGMDELYATNFSLLKQAGDVEENPGPIGTGFPFDPHYVEVLGERMHY
VDVGPRDGTPVLFLHGNPTSSYVWRNIIPHVAPTHRCIAPDLIGMGKSDKPDLGYFFDDHVRFMDAFIEALGLEE
VVLVIHDWGSALGFHWAKRNPERVKGIAFMEFIRPIPTWDEWPKFARKTFQAFRTKKVGRKLIIDQNVFIEGTLP
MGVVRPLTEVEMDHYREPFLNPVDREPLWRFPNELPIAGEPANIVALVEEYMDWLHQSPVPKLLFWGTPGVLIPP
AEAARLAKSLPNCKAVDIGPGLNLLQEDNPDLIGSEIARWLSTLEISGMVSKGEAVIKEFMRFKVHMEGSMNGHE
FEIEGEGEGRPYEGTQTAKLKVTKGGPLPFSWDILSPQFMYGSRAFIKHPADIPDYYKQSFPEGFKWERVMNFED
GGAVTVTQDTSLEDGTLIYKVKLRGTNFPPDGPVMQKKTMGWEASTERLYPEDGVLKGDIKMALRLKDGGRYLAD
FKTTYKAKKPVQMPGAYNVDRKLDITSHNEDYTVVEQYERSEGRHSTGGMDELYK

> DNA Sequence

ATGATCAGTCTGATTGCGGCGTTAGCGGTAGATCGCGTTATCGGCATGGAAAACGCCATGCCGTGGAACCTGCCT
GCCGATCTCGCCTGGTTTAAACGCAACACCTTAAATAAACCCGTGATTATGGGCCGCCATACCTGGGAATCAATC
GGTCGTCCGTTGCCAGGACGCAAAAATATTATCCTCAGCAGTCAACCGGGTACGGACAAAAAAAAGAAAAAGAAA
GATCGCGTAACGTGGGTGAAGTCGGTGGATGAAGCCATCGCGGCGTGTGGTGACGTACCAGAAATCATGGTGATT
GGCGGCGGTCGCGTTTATGAACAGTTCTTGCCAAAAGCGCAAAAACTGTATCTGACGCATATCGACGCAGAAGTG
GAAGGCGACACCCATTTCCCGGATTACGAGCCGGATGACTGGGAATCGGTATTCAGCGAATTCCACGATGCTGAT
GCGCAGAACTCTCACAGCTATTGCTTTGAGATTCTGGAGCGGCGGAGCGGCTCCGGGGATCCACCGGTCGCCACC
ATGGTGAGCAAGGGCGAGGAGGATAACATGGCCTCTCTCCCAGCGACACATGAGTTACACATCTTTGGCTCCATC
AACGGTGTGGACTTTGACATGGTGGGTCAGGGCACCGGCAATCCAAATGATGGTTATGAGGAGTTAAACCTGAAG
TCCACCAAGGGTGACCTCCAGTTCTCCCCCTGGATTCTGGTCCCTCATATCGGGTATGGCTTCCATCAGTACCTG
CCCTACCCTGACGGGATGTCGCCTTTCCAGGCCGCCATGGTAGATGGCTCCGGATACCAAGTCCATCGCACAATG
CAGTTTGAAGATGGTGCCTCCCTTACTGTTAACTACCGCTACACCTACGAGGGAAGCCACATCAAAGGAGAGGCC
CAGGTGAAGGGGACTGGTTTCCCTGCTGACGGTCCTGTGATGACCAACTCGCTGACCGCTGCGGACTGGTGCAGG
TCGAAGAAGACTTACCCCAACGACAAAACCATCATCAGTACCTTTAAGTGGAGTTACACCACTGGAAATGGCAAG
CGCTACCGGAGCACTGCGCGGACCACCTACACCTTTGCCAAGCCAATGGCGGCTAACTATCTGAAGAACCAGCCG
ATGTACGTGTTCCGTAAGACGGAGCTCAAGCACTCCAAGACCGAGCTCAACTTCAAGGAGTGGCAAAAGGCCTTT
ACCGATGTGATGGGCATGGACGAGCTGTACGCCACAAACTTCTCTCTGCTAAAGCAAGCAGGTGATGTTGAAGAA
AACCCCGGGCCTATTGGCACCGGTTTTCCGTTTGATCCGCATTATGTTGAAGTTCTGGGTGAACGTATGCATTAT
GTGGATGTTGGTCCGCGTGATGGTACACCGGTTCTGTTTCTGCATGGTAATCCGACCAGCAGCTATGTTTGGCGT
AACATTATTCCGCATGTTGCACCGACCCATCGTTGTATTGCACCGGATCTGATTGGTATGGGTAAAAGCGATAAA
CCTGATCTGGGCTATTTTTTCGATGATCATGTGCGTTTTATGGACGCCTTTATTGAAGCACTGGGTCTGGAAGAA
GTTGTGCTGGTTATTCATGATTGGGGTAGCGCACTGGGTTTTCATTGGGCAAAACGTAATCCGGAACGTGTTAAA
GGTATTGCCTTTATGGAATTTATTCGTCCGATTCCGACCTGGGATGAATGGCCGAAATTTGCACGTAAAACCTTT
CAGGCATTTCGCACCAAAAAAGTTGGTCGCAAACTGATTATTGACCAGAACGTTTTTATCGAAGGCACCCTGCCG
ATGGGTGTTGTTCGTCCGCTGACCGAAGTTGAAATGGATCATTATCGTGAACCGTTTCTGAATCCGGTTGATCGC
GAACCGCTGTGGCGTTTTCCGAATGAACTGCCGATTGCCGGTGAACCTGCAAATATTGTTGCACTGGTTGAAGAG
TATATGGATTGGCTGCATCAGAGTCCGGTTCCGAAACTGCTGTTTTGGGGCACACCGGGTGTTCTGATTCCGCCT
GCAGAAGCAGCACGTCTGGCAAAAAGCCTGCCGAATTGTAAAGCAGTTGATATTGGTCCGGGTCTGAATCTGCTG
CAAGAAGATAATCCAGATCTGATCGGTAGTGAAATTGCACGTTGGCTGAGCACCCTGGAAATTAGCGGCATGGTG
AGCAAGGGCGAGGCAGTGATCAAGGAGTTCATGCGGTTCAAGGTGCACATGGAGGGCTCCATGAACGGCCACGAG
TTCGAGATCGAGGGCGAGGGCGAGGGCCGCCCCTACGAGGGCACCCAGACCGCCAAGCTGAAGGTGACCAAGGGT
GGCCCCCTGCCCTTCTCCTGGGACATCCTGTCCCCTCAGTTCATGTACGGCTCCAGGGCCTTCAUCAAGCACCCC

GCCGACATCCCCGACTACTATAAGCAGTCCTTCCCCGAGGGCTTCAAGTGGGAGCGCGTGATGAACTTCGAGGAC
GGCGGCGCCGTGACCGTGACCCAGGACACCTCCCTGGAGGACGGCACCCTGATCTACAAGGTGAAGCTCCGCGGC
ACCAACTTCCCTCCTGACGGCCCCGTAATGCAGAAGAAGACAATGGGCTGGGAAGCGTCCACCGAGCGGTTGTAC
CCCGAGGACGGCGTGCTGAAGGGCGACATTAAGATGGCCCTGCGCCTGAAGGACGGCGGCCGCTACCTGGCGGAC
TTCAAGACCACCTACAAGGCCAAGAAGCCCGTGCAGATGCCCGGCGCCTACAACGTCGACCGCAAGTTGGACATC
ACCTCCCACAACGAGGACTACACCGTGGTGGAACAGTACGAACGCTCCGAGGGCCGCCACTCCACCGGCGGCATG
GACGAGCTGTACAAGTAG

^{iK6}eDHFR 6x internal Lysine Linker mNeonGreen P2A HOB mScarlet T74I mutation

pCMV-^{iK6}eDHFR-mEGFP-BCR$^{Rho\ GAP}$

> Amino acid sequence

MISLIAALAVDRVIGMENAMPWNLPADLAWFKRNTLNKPVIMGRHTWESIGRPLPGRKNIILSSQPGTDKKKKKK
DRVTWVKSVDEAIAACGDVPEIMVIGGGRVYEQFLPKAQKLYLTHIDAEVEGDTHFPDYEPDDWESVFSEFHDAD
AQNSHSYCFEILERRSGSGDPPVATMMVSKGEELFTGVVPILVELDGDVNGHKFSVSGEGEGDATYGKLTLKFIC
TTGKLPVPWPTLVTTLTYGVQCFSRYPDHMKQHDFFKSAMPEGYVQERTIFFKDDGNYKTRAEVKFEGDTLVNRI
ELKGIDFKEDGNILGHKLEYNYNSHNVYIMADKQKNGIKVNFKIRHNIEDGSVQLADHYQQNTPIGDGPVLLPDN
HYLSTQSKLSKDPNEKRDHMVLLEFVTAAGITLGMDELYKSGLRSRQGSGAGSGAGSGAGSGAGSGAPRARIPLD
PQALQDRDWQRTVIAMNGIEVKLSVKFNSREFSLKRMPSRKQTGVFGVKIAVVTKRERSKVPYIVRQCVEEIERR
GMEEVGIYRVSGVATDIQALKAAFDVNNKDVSVMMSEMDVNAIAGTLKLYFRELPEPLFTDEFYPNFAEGIALSD
PVAKESCMLNLLLSLPEANLLTFLFLLDHLKRVAEKEAVNKMSLHNLATVFGPTLLRPSEKESKLPANPSQPITM
TDSWSLEVMSQVQVLLYFLQLEAIPAPDSKRQSILFSTEV

> DNA Sequence

ATGATCAGTCTGATTGCGGCGTTAGCGGTAGATCGCGTTATCGGCATGGAAAACGCCATGCCGTGGAACCTGCCT
GCCGATCTCGCCTGGTTTAAACGCAACACCTTAAATAAACCCGTGATTATGGGCCGCCATACCTGGGAATCAATC
GGTCGTCCGTTGCCAGGACGCAAAAATATTATCCTCAGCAGTCAACCGGGTACGGACAAAAAAAAGAAAAAGAAA
GATCGCGTAACGTGGGTGAAGTCGGTGGATGAAGCCATCGCGGCGTGTGGTGACGTACCAGAAATCATGGTGATT
GGCGGCGGTCGCGTTTATGAACAGTTCTTGCCAAAAGCGCAAAAACTGTATCTGACGCATATCGACGCAGAAGTG
GAAGGCGACACCCATTTCCCGGATTACGAGCCGGATGACTGGGAATCGGTATTCAGCGAATTCCACGATGCTGAT
GCGCAGAACTCTCACAGCTATTGCTTTGAGATTCTGGAGCGGCGGAGCGGCTCCGGGGATCCACCGGTCGCCACC
ATGATGGTGAGCAAGGGCGAGGAGCTGTTCACCGGGGTGGTGCCCATCCTGGTCGAGCTGGACGGCGACGTAAAC
GGCCACAAGTTCAGCGTGTCCGGCGAGGGCGAGGGCGATGCCACCTACGGCAAGCTGACCCTGAAGTTCATCTGC
ACCACCGGCAAGCTGCCCGTGCCCTGGCCCACCCTCGTGACCACCCTGACCTACGGCGTGCAGTGCTTCAGCCGC
TACCCCGACCACATGAAGCAGCACGACTTCTTCAAGTCCGCCATGCCCGAAGGCTACGTCCAGGAGCGCACCATC
TTCTTCAAGGACGACGGCAACTACAAGACCCGCGCCGAGGTGAAGTTCGAGGGCGACACCCTGGTGAACCGCATC
GAGCTGAAGGGCATCGACTTCAAGGAGGACGGCAACATCCTGGGGCACAAGCTGGAGTACAACTACAACAGCCAC
AACGTCTATATCATGGCCGACAAGCAGAAGAACGGCATCAAGGTGAACTTCAAGATCCGCCACAACATCGAGGAC
GGCAGCGTGCAGCTCGCCGACCACTACCAGCAGAACACCCCCATCGGCGACGGCCCCGTGCTGCTGCCCGACAAC
CACTACCTGAGCACCCAGTCCAAACTGAGCAAAGACCCCAACGAGAAGCGCGATCACATGGTCCTGCTGGAGTTC
GTGACCGCCGCCGGGATCACTCTCGGCATGGACGAGCTGTACAAGAGCGGCCTGCGCAGCCGCCAGGGCAGTGGT
GCTGGCTCTGGTGCTGGTAGTGGCGCTGGTTCCGGTGCGGGCAGCGGCGCGCCGCGCGCGAGGATCCCCCTGGAC
CCGCAGGCCCTGCAGGACAGAGACTGGCAGCGCACCGTCATCGCCATGAATGGGATCGAAGTAAAGCTCTCGGTC
AAGTTCAACAGCAGGGAGTTCAGCTTGAAGAGGATGCCGTCCCGAAAACAGACAGGGGTCTTCGGAGTCAAGATT
GCTGTGGTCACCAAGAGAGAGAGGTCCAAGGTGCCCTACATCGTGCGCCAGTGCGTGGAGGAGATCGAGCGCCGA
GGCATGGAGGAGGTGGGCATCTACCGCGTGTCCGGTGTGGCCACGGACATCCAGGCACTGAAGGCAGCCTTCGAC
GTCAATAACAAGGACGTGTCGGTGATGATGAGCGAGATGGACGTGAACGCCATCGCAGGCACGCTGAAGCTGTAC
TTCCGTGAGCTGCCCGAGCCCCTCTTCACTGACGAGTTCTACCCCAACTTCGCAGAGGGCATCGCTCTTTCAGAC
CCGGTTGCAAAGGAGAGCTGCATGCTCAACCTGCTGCTGTCCCTGCCGGAGGCCAACCTGCTCACCTTCCTTTTC
CTTCTGGACCACCTGAAAAGGGTGGCAGAGAAGGAGGCAGTCAATAAGATGTCCCTGCACAACCTCGCCACGGTC
TTTGGCCCCACGCTGCTCCGGCCCTCCGAGAAGGAGAGCAAGCTCCCTGCCAACCCCAGCCAGCCTATCACCATG
ACTGACAGCTGGTCCTTGGAGGTCATGTCCCAGGTCCAGGTGCTGCTGTACTTCCTGCAGCTGGAGGCCATCCCT
GCCCCGGACAGCAAGAGACAGAGCATCCTGTTCTCCACCGAAGTCTAG

^{iK6}eDHFR 6x internal Lysine Linker mEGFP Linker BCR$^{Rho\ GAP}$

pCMV-HOB-mScarlet-Tiam1$^{DH/PH}$

> Amino acid sequence

MIGTGFPFDPHYVEVLGERMHYVDVGPRDGTPVLFLHGNPTSSYVWRNIIPHVAPTHRCIAPDLIGMGKSDKPDL
GYFFDDHVRFMDAFIEALGLEEVVLVIHDWGSALGFHWAKRNPERVKGIAFMEFIRPIPTWDEWPKFARKTFQAF
RTKKVGRKLIIDQNVFIEGTLPMGVVRPLTEVEMDHYREPFLNPVDREPLWRFPNELPIAGEPANIVALVEEYMD
WLHQSPVPKLLFWGTPGVLIPPAEAARLAKSLPNCKAVDIGPGLNLLQEDNPDLIGSEIARWLSTLEISGMVSKG
EAVIKEFMRFKVHMEGSMNGHEFEIEGEGEGRPYEGTQTAKLKVTKGGPLPFSWDILSPQFMYGSRAFTKHPADI
PDYYKQSFPEGFKWERVMNFEDGGAVTVTQDTSLEDGTLIYKVKLRGTNFPPDGPVMQKKTMGWEASTERLYPED
GVLKGDIKMALRLKDGGRYLADFKTTYKAKKPVQMPGAYNVDRKLDITSHNEDYTVVEQYERSEGRHSTGGMDEL
YKSGLRSRQGSGAGSGAGSGAGSGAGSGAPRAMNPSDQSPSPQDSTGPQLATMRQLSDADKLRKVICELLETERT
YVKDLNCLMERYLKPLQKETFLTQDELDVLFGNLTEMVEFQVEFLKTLEDGVRLVPDLEKLEKVDQFKKVLFSLG
GSFLYYADRFKLYSAFCASHTKVPKVLVKAKTDTAFKAFLDAQNPKQQHSSTLESYLIKPIQRILKYPLLLRELF
ALTDAESEEHYHLDVAIKTMNKVASHINEMQKIHEEFGAVFDQLIAEQTGGKKEVADLSMGDLLLHTTVIWLNPP
ASLGKWKKEPELAAFVFKTAVVLVYKDGSKQKKKLVGSHRLSIYEDWDPFRFRHMIPTEALQVRALASADAEANA
VCEIVHVKSESEGRPERVFHLCCSSPESRKDFLKAVHSILRDKHRRQLLKTESLPSSQQYVPFGGKRLCALKGAR
PAMSRAVSAPSKSLGRRRRRLARNRFTIDSDAVSASSPEKESQQPPGGGDTDRWVEEQFDLAQYEEQDDIKETDI
LSDDDEFCESVKGASVDRDLQERLQATSISQRERGRKTLDSHASRMAQLKKQAALSGINGGLESASEEVIWVRRE
DFAPSRKLNTEI

> DNA Sequence

ATGATTGGCACCGGTTTTCCGTTTGATCCGCATTATGTTGAAGTTCTGGGTGAACGTATGCATTATGTGGATGTT
GGTCCGCGTGATGGTACACCGGTTCTGTTTCTGCATGGTAATCCGACCAGCAGCTATGTTTGGCGTAACATTATT
CCGCATGTTGCACCGACCCATCGTTGTATTGCACCGGATCTGATTGGTATGGGTAAAAGCGATAAACCTGATCTG
GGCTATTTTTTCGATGATCATGTGCGTTTTATGGACGCCTTTATTGAAGCACTGGGTCTGGAAGAAGTTGTGCTG
GTTATTCATGATTGGGGTAGCGCACTGGGTTTTCATTGGGCAAAACGTAATCCGGAACGTGTTAAAGGTATTGCC
TTTATGGAATTTATTCGTCCGATTCCGACCTGGGATGAATGGCCGAAATTTGCACGTAAAACCTTTCAGGCATTT
CGCACCAAAAAAGTTGGTCGCAAACTGATTATTGACCAGAACGTTTTTATCGAAGGCACCCTGCCGATGGGTGTT
GTTCGTCCGCTGACCGAAGTTGAAATGGATCATTATCGTGAACCGTTTCTGAATCCGGTTGATCGCGAACCGCTG
TGGCGTTTTCCGAATGAACTGCCGATTGCCGGTGAACCTGCAAATATTGTTGCACTGGTTGAAGAGTATATGGAT
TGGCTGCATCAGAGTCCGGTTCCGAAACTGCTGTTTTGGGGCACACCGGGTGTTCTGATTCCGCCTGCAGAAGCA
GCACGTCTGGCAAAAAGCCTGCCGAATTGTAAAGCAGTTGATATTGGTCCGGGTCTGAATCTGCTGCAAGAAGAT
AATCCAGATCTGATCGGTAGTGAAATTGCACGTTGGCTGAGCACCCTGGAAATTAGCGGCATGGTGAGCAAGGGC
GAGGCAGTGATCAAGGAGTTCATGCGGTTCAAGGTGCACATGGAGGGCTCCATGAACGGCCACGAGTTCGAGATC
GAGGGCGAGGGCGAGGGCCGCCCCTACGAGGGCACCCAGACCGCCAAGCTGAAGGTGACCAAGGGTGGCCCCCTG
CCCTTCTCCTGGGACATCCTGTCCCCTCAGTTCATGTACGGCTCCAGGGCCTTCACCAAGCACCCCGCCGACATC
CCCGACTACTATAAGCAGTCCTTCCCCGAGGGCTTCAAGTGGGAGCGCGTGATGAACTTCGAGGACGGCGGCGCC
GTGACCGTGACCCAGGACACCTCCCTGGAGGACGGCACCCTGATCTACAAGGTGAAGCTCCGCGGCACCAACTTC
CCTCCTGACGGCCCCGTAATGCAGAAGAAGACAATGGGCTGGGAAGCGTCCACCGAGCGGTTGTACCCCGAGGAC
GGCGTGCTGAAGGGCGACATTAAGATGGCCCTGCGCCTGAAGGACGGCGGCCGCTACCTGGCGGACTTCAAGACC
ACCTACAAGGCCAAGAAGCCCGTGCAGATGCCCGGCGCCTACAACGTCGACCGCAAGTTGGACATCACCTCCCAC
AACGAGGACTACACCGTGGTGGAACAGTACGAACGCTCCGAGGGCCGCCACTCCACCGGCGGCATGGACGAGCTG
TACAAGTCCGGACTCAGATCTCGACAAGGTAGTGGTGCTGGCTCTGGTGCTGGTAGTGGCGCTGGTTCCGGTGCT
GGCTCTGGCGCGCCTCGAGCAATGAACCCCTCTGACCAGAGCCCATCTCCTCAGGACTCCACGGGGCCTCAGCTG
GCGACCATGAGACAACTCTCGGATGCAGATAAGCTGCGCAAGGTGATCTGCGAGCTCCTGGAGACGGAGCGCACC
TACGTGAAGGATTTAAACTGTCTTATGGAGAGATACCTAAAGCCTCTTCAAAAAGAAACTTTTCTCACCCAGGAT
GAGCTTGACGTGCTTTTTGGAAATTTAACGGAAATGGTAGAGTTTCAAGTAGAATTCCTTAAAACTCTAGAAGAT
GGAGTGAGACTGGTACCTGATTTGGAAAAGCTTGAGAAGGTTGATCAATTTAAGAAAGTGCTGTTCTCTCTGGGG
GGATCATTCCTGTATTATGCTGACCGCTTCAAGCTCTACAGTGCCTTCTGCGCCAGCCACACAAAAGTTCCCAAG
GTCCTGGTGAAAGCCAAGACAGACACGGCTTTCAAGGCATTCTTGGATGCCCAGAACCCGAAGCAGCAGCACTCA

TCCACGCTGGAGTCGTACCTCATCAAGCCCATCCAGAGGATCCTCAAGTACCCACTTCTGCTCAGGGAGCTGTTC
GCCCTGACCGATGCGGAGAGCGAGGAGCACTACCACCTGGACGTGGCCATCAAGACCATGAACAAGGTTGCCAGT
CACATCAATGAGATGCAGAAAATCCATGAAGAGTTTGGGGCTGTGTTTGACCAGCTGATTGCTGAACAGACTGGT
GGGAAAAAAGAGGTTGCAGATCTGAGCATGGGAGACCTGCTTTTGCACACTACCGTGATCTGGCTGAACCCGCCG
GCCTCGCTGGGCAAGTGGAAAAAGGAACCAGAGTTGGCAGCATTCGTCTTCAAAACTGCTGTGGTCCTTGTGTAT
AAAGATGGTTCCAAACAGAAGAAGAAACTTGTAGGATCTCACAGGCTTTCCATTTATGAGGACTGGGACCCCTTC
AGATTTCGACACATGATCCCCACGGAAGCGCTGCAGGTTCGAGCTTTGGCGAGTGCAGATGCAGAGGCAAATGCC
GTGTGTGAAATTGTCCATGTAAAATCCGAGTCTGAAGGGAGGCCGGAGAGGGTCTTTCACTTGTGCTGCAGCTCC
CCAGAGAGCCGAAAGGATTTCCTAAAGGCTGTGCATTCAATCCTGCGTGATAAGCACAGAAGACAGCTCCTCAAA
ACCGAGAGCCTTCCCTCATCCCAGCAATATGTCCCTTTTGGAGGCAAAAGATTGTGTGCACTGAAGGGGGCCAGG
CCGGCCATGAGCAGGGCAGTGTCTGCCCCAAGCAAGTCTCTTGGGAGGAGGAGGCGGCGGCTGGCTCGAAACAGG
TTTACCATTGATTCTGATGCCGTCTCCGCAAGCAGCCCGGAGAAAGAGTCCCAGCAGCCCCCCGGTGGCGGGGAC
ACTGACCGATGGGTAGAGGAGCAGTTTGATCTTGCTCAGTATGAGGAGCAAGATGACATCAAGGAGACAGACATC
CTCAGTGACGATGATGAGTTCTGTGAGTCCGTGAAGGGTGCCTCAGTGGACAGAGACCTGCAGGAGCGGCTTCAG
GCCACCTCCATCAGTCAGCGGGAAAGAGGCCGGAAAACCCTGGATAGTCACGCGTCCCGCATGGCACAGCTCAAG
AAGCAAGCTGCCCTGTCGGGGATCAATGGAGGCCTGGAGAGCGCAAGCGAGGAAGTCATTTGGGTTAGGCGTGAA
GACTTTGCCCCCTCCAGGAAACTGAACACTGAGATCTGATAG

HOB mScarlet Linker Tiam1$^{DH/PH}$

Publication bibliography

Ando, Tomomi; Tsukiji, Shinya; Tanaka, Tsutomu; Nagamune, Teruyuki (2007): Construction of a small-molecule-integrated semisynthetic split intein for in vivo protein ligation. In *Chemical communications (Cambridge, England)* (47), pp. 4995–4997. DOI: 10.1039/B712843F.

Ballister, Edward R.; Aonbangkhen, Chanat; Mayo, Alyssa M.; Lampson, Michael A.; Chenoweth, David M. (2014): Localized light-induced protein dimerization in living cells using a photocaged dimerizer. In *Nat Commun* 5 (1), p. 5475. DOI: 10.1038/ncomms6475.

McCullock, Tyler W.; MacLean, David M.; Kammermeier, Paul J. (2020): Comparing the performance of mScarlet-I, mRuby3, and mCherry as FRET acceptors for mNeonGreen. In *PLOS ONE* 15 (2), e0219886. DOI: 10.1371/journal.pone.0219886.

Nakamura, Akinobu; Oki, Choji; Kato, Kenya; Fujinuma, Satoko; Maryu, Gembu; Kuwata, Keiko et al. (2020a): Engineering Orthogonal, Plasma Membrane-Specific SLIPT Systems for Multiplexed Chemical Control of Signaling Pathways in Living Single Cells. In *ACS Chemical Biology* 15 (4), pp. 1004–1015. DOI: 10.1021/acschembio.0c00024.

Nakamura, Akinobu; Oki, Choji; Sawada, Shunsuke; Yoshii, Tatsuyuki; Kuwata, Keiko; Rudd, Andrew K. et al. (2020b): Designer Palmitoylation Motif-Based Self-Localizing Ligand for Sustained Control of Protein Localization in Living Cells and Caenorhabditis elegans. In *ACS Chemical Biology* 15 (4), pp. 837–843. DOI: 10.1021/acschembio.0c00014.

Spiering, Désirée; Bravo-Cordero, Jose Javier; Moshfegh, Yasmin; Miskolci, Veronika; Hodgson, Louis (2013): Quantitative ratiometric imaging of FRET-biosensors in living cells. In *Methods in cell biology* 114, pp. 593–609. DOI: 10.1016/B978-0-12-407761-4.00025-7.

Yoshii, Tatsuyuki; Oki, Choji; Watahiki, Rei; Nakamura, Akinobu; Tahara, Kai; Kuwata, Keiko et al. (2021): Chemo-optogenetic Protein Translocation System Using a Photoactivatable Self-Localizing Ligand. In *ACS Chemical Biology* 16 (8), pp. 1557–1565. DOI: 10.1021/acschembio.1c00416.
